# Laparoscopic adjustable gastric banding with liraglutide in adults with obesity and type 2 diabetes (GLIDE): a pilot randomised placebo controlled trial

**DOI:** 10.1038/s41366-023-01368-4

**Published:** 2023-09-11

**Authors:** Claudia Coelho, Laurence J. Dobbie, James Crane, Abdel Douiri, Annastazia E. Learoyd, Olanike Okolo, Spyros Panagiotopoulos, Dimitri J. Pournaras, Sasindran Ramar, Francesco Rubino, Rishi Singhal, Carel W. le Roux, Shahrad Taheri, Barbara McGowan

**Affiliations:** 1 Department of Diabetes and Endocrinology, Guy’s and St Thomas’ NHS Foundation Trust, London, UK; 2 Department of Endocrinology, King’s College Hospital NHS Foundation Trust, London, UK; 3 School of Life Course and Population Sciences, Faculty of Life Sciences & Medicine, King College London, London, UK; 4 Department of Minimal Access Surgery, King’s College Hospital NHS Foundation Trust, London, UK; 5 Department of Upper GI and Bariatric/Metabolic Surgery, North Bristol NHS Trust, Southmead Hospital, Bristol, UK; 6 Department of Diabetes, School of Life Course Sciences, King’s College London, London, UK; 7 Upper GI Unit at Heart of England, NHS Foundation Trust, Birmingham, UK; 8 Diabetes Complications Research Centre, University College Dublin, Dublin, Ireland UK; 9 Department of Medicine, Weill Cornell Medicine Qatar, Doha, Qatar

**Keywords:** Randomized controlled trials, Drug development, Phase IV trials

## Abstract

**Introduction:**

Obesity drives type 2 diabetes (T2DM) development. Laparoscopic adjustable gastric banding (LAGB) has lower weight reduction than other bariatric procedures. Liraglutide, a GLP-1 receptor agonist, improves weight and glycaemic control in patients with T2DM. This study aimed to determine the efficacy and safety of liraglutide 1.8 mg in participants undergoing LAGB.

**Methods:**

GLIDE, a pilot randomised, double-blind, placebo-controlled trial, evaluated LAGB with either liraglutide 1.8 mg or placebo in participants with T2DM and obesity. Participants were randomised (1:1) to 6-months therapy post-LAGB, with further 6 months off-treatment follow-up. The primary outcome was change in HbA1c from randomisation to the end of treatment, secondary outcomes included body weight change. A sample size of 58 (29 per group) had 80% power to detect a 0.6% difference in HbA1c between groups.

**Results:**

Twenty-seven participants were randomised to liraglutide (*n* = 13) or placebo (*n* = 14). Multivariate analysis showed no difference between placebo and liraglutide arms in HbA1c at 6 months (HbA1c:0.2 mmol/mol, −11.3, 11.6, *p* = 0.98) however, at 12 months HbA1c was significantly higher in the liraglutide arm (HbA1c:10.9 mmol/mol, 1.1, 20.6, *p* = 0.032). There was no difference between arms in weight at 6 months (BW:2.0 kg, −4.2, 8.1, *p* = 0.50), however, at 12 months weight was significantly higher in the liraglutide arm (BW:8.2 kg, 1.6, 14.9, *p* = 0.02). There were no significant differences in adverse events between groups.

**Conclusions:**

Our pilot data suggest no additional improvement in glycaemic control or BW with LAGB and liraglutide therapy. However, this trial was significantly underpowered to detect a significant change in the primary or secondary outcomes. Further trials are needed to investigate whether GLP-1 agonists, and particularly with more effective weekly agents (i.e. semaglutide or tirzepatide), are of benefit following metabolic surgery.

**Clinical trial registration:**

EudraCT number 2015-005402-11.

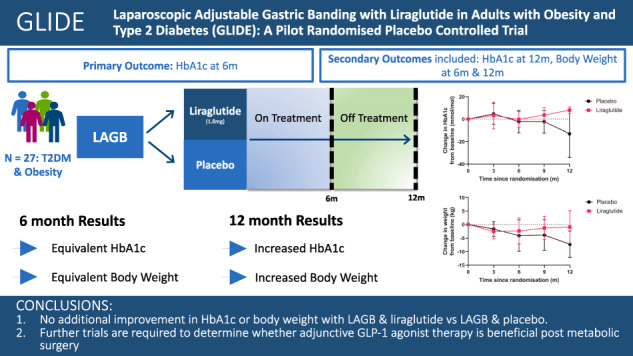

## Introduction

Obesity is defined as excess adiposity causing a deterioration in health [1]. Obesity is implicated in the development of cardiometabolic complications including type 2 diabetes (T2DM), coronary heart disease, non-alcoholic fatty liver disease and certain cancers [2, 3]. Currently, 25% of adults in the United Kingdom are living with obesity and the rise in obesity has significantly increased the number of patients with T2DM [4–6]. Obesity management involves lifestyle interventions [7], pharmacotherapy [8, 9] and bariatric surgery [10]. Bariatric surgery, in combination with additional pharmacotherapy, may be a therapeutic option to address diabetes and obesity simultaneously [11].

Bariatric surgical procedures include laparoscopic adjustable gastric banding (LAGB), laparoscopic sleeve gastrectomy (LSG) and Roux-en-Y gastric bypass (RYGB) [6, 12–15]. Bariatric surgery can result in remission of T2DM, with a large meta-analysis (*n* = 22094) reporting more frequent resolution of diabetes in patients undergoing RYGB (83.8%) than LAGB (47.8%) [16]. A likely mechanism underpinning superiority of RYGB over LAGB on diabetes remission is the enhanced gut hormone secretion, including glucagon-like peptide-1 (GLP-1) and peptide tyrosine tyrosine [17, 18]. However, RYGB has higher operative mortality (0.09% RYGB vs 0.03% LAGB) and is associated with longer hospital stay, greater morbidity and vitamin deficiency [19]. Therefore, interventions which improve diabetes and weight loss alongside minimising risks and associated morbidity are of interest. Potentially, pharmacological enhancement of incretin secretion could bridge the gap between RYGB and LAGB on body weight and diabetes-related outcomes.

There is evidence underpinning adjunctive GLP-1 therapy post-bariatric surgery [20]. Physiologically, RYGB improves glycaemic control and gut hormone responses (GLP-1 and pancreatic polypeptide) post-operatively compared to LAGB [21]. Similarly, in individuals living with obesity and T2DM, RYGB causes a greater enhancement of release of GLP-1 than LAGB [17]. Clinically, a systematic review (*n* = 7971 patients) reported that T2DM remission rates were greater following RYGB (66.7% RYGB vs 28.6% LAGB remission rate) [10]. Therefore, GLP-1 agonist therapy may be a potential therapeutic adjunct in patients undergoing LAGB with diabetes, potentially improving glycaemic control, weight loss and diabetes remission rate. Liraglutide (Victoza®, Novo Nordisk) is a GLP-1 analogue emulating the human GLP-1 hormone [22, 23]. The “LEAD (Liraglutide Effect and Action Diabetes) program” reported that liraglutide significantly reduced weight and improved glycated haemoglobin (HbA1c) compared to placebo/standard of care [24–31]. This study aimed to determine whether the addition of liraglutide 1.8 mg (Victoza®) to LAGB leads to clinically significant improvements in HbA1c compared to LAGB alone. We hypothesised that LAGB and Liraglutide 1.8 mg would improve glycaemic control to a greater degree than LAGB alone.

## Methods

### Trial design and oversight

We conducted a randomised, double-blind, placebo-controlled trial at three sites in the United Kingdom. The study was prospectively registered with EudraCT (Registration Number: 2015-005402-11) and overseen by the sponsor, King’s Health Partners. The study was conducted in adherence with the Good Clinical Practice Guidelines and the principles of the Declaration of Helsinki. The protocol was approved by London—Westminster Research Ethics Committee (REC Reference: 16/LO/1144).

### Participants

We recruited adults (age 18–70) with a body mass index (BMI) of 30–50 kg/m^2^ and T2DM (HbA1c ≥ 48 mmol/mol and <97 mmol/mol at or before screening). Participants were recruited from outpatient weight management services at participating centres. All participants provided written informed consent. Key exclusion criteria were type 1 diabetes, history of delayed gastric emptying, diet-controlled T2DM, pregnancy or breastfeeding, history of pancreatitis and personal or family history of thyroid cancer or multiple endocrine neoplasia. Inclusion and exclusion criteria are provided in Supplementary Table 1.

### Procedures

**Fig. 1:**
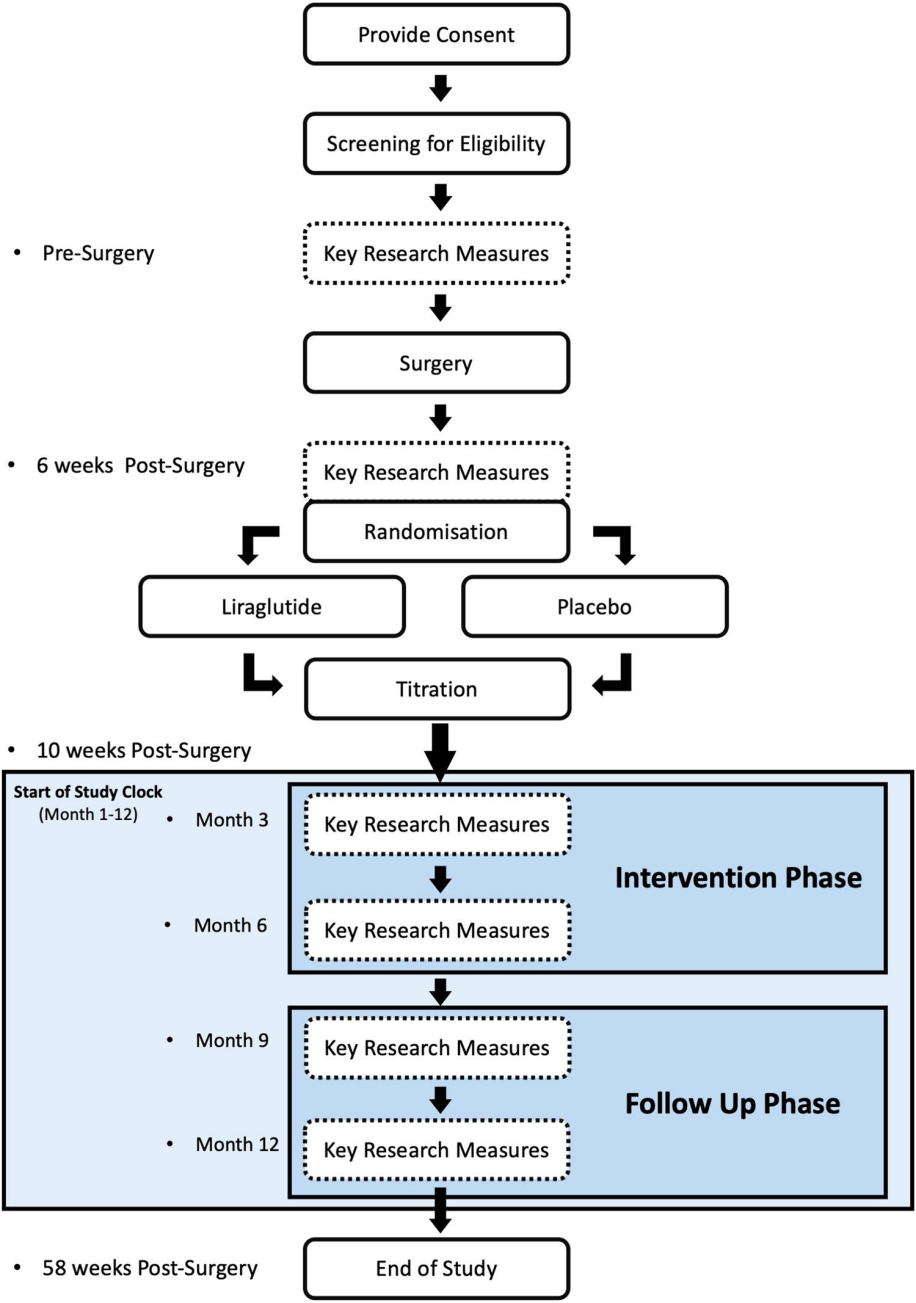
Trial Flow Chart.

Participants underwent insertion of LAGB. Participants were randomised in a 1:1 ratio to either subcutaneous liraglutide 1.8 mg (Victoza) or placebo once daily within 6 weeks of surgery. Treatment allocation was fully concealed. Liraglutide was titrated as recommended to a maximum tolerated dose of 1.8 mg. The liraglutide/placebo intervention duration for each participant was 6 months (including the titration phase). Participants were followed up for 12 months and attended seven visits in total (Fig. 1).

### Laparoscopic adjustable band procedures and follow up

This was a pragmatic trial. Although no specific surgical approach to LAGB placement was specified, as far as possible, standardisation in clinical approach was agreed upon amongst participating centres. The band’s type, size and specifications and operative procedure were documented in the clinical notes. When the gastric band was reviewed, this was recorded in the Case Report Form (CRF) along with any adjustments’ date(s). The participants’ follow-up was carried out according to clinical protocols, and need, including appropriate nutritional and hydration advice and band-fill adjustments by specialist dieticians or other appropriately trained staff blinded to the study arm, depending on local policy.

### Randomisation

Randomisation was carried out by the King’s Clinical Trials Unit through a web-based randomisation software utilising a minimisation algorithm. Minimisation, including a random component (0.8), was carried out to protect the balance between groups. Factors used in minimisation were centre, BMI (≤42 and >42), use of insulin and diabetes duration (≤5 and >5 years). Participants were randomised to liraglutide or placebo. Once a participant was randomised, the system automatically generated emails to key staff within the study. Unblinded e-mails sent to site pharmacies alerted them to a participant’s treatment arm. The investigational medicinal product (IMP) was packaged with a unique dispensing unit number (DUN), and the pharmacy had a copy of the Total DUN List (TDL) which detailed the identity of each DUN. The pharmacy department used email and TDL to cross-check the trial prescription to ensure that the correct medication was being dispensed to the correct participant. The TDL was not available to any other research team members who remained blinded. Apart from pharmacy staff, all other site staff were blinded to the treatment arm of the participants.

### Endpoints

The primary endpoint was glycaemic control (change in HbA1c) as a measure of the impact of the addition of liraglutide 1.8 mg once daily on the clinical efficacy of LAGB in treating T2DM between randomisation and 6 months.

Secondary endpoints of interest (Change between randomisation and 6–12 months)

- **Diabetes control**
  ∘ Percentage of participants with remission of diabetes at 12 months (HbA1c < 48 mmol/mol)
  ∘ Homeostatic model assessment – insulin resistance (HOMA-IR)
  ∘ Hypoglycaemic episodes

- **Body Composition**
  ∘ Body Weight
  ∘ BMI
  ∘ Waist circumference
  ∘ Neck circumference
  ∘ Bioimpedance

- **Cardiovascular Disease Risk**
  ∘ Systolic Blood Pressure
  ∘ Diastolic Blood Pressure
  ∘ Lipid Profile – Total Cholesterol, HDL, LDL, TG
- **Physical Activity Levels** – GPAQ questionnaire
- **Quality of Life and Psychological Measures** – IWQOL-Lite, HAS, EQ-5D-5L, EuroQol

### Statistics

A statistical plan was drafted a priori and approved by the ethics committee. A sample size of 58 (29 per group) had 80% power to detect a 0.6% difference in HbA1c (above the minimum clinically important difference of 0.5%) between groups. This accounted for 20% drop-out or loss to follow-up. All analyses followed the intention-to-treat principle and significance was taken at the 5% level. The statistician was blinded to treatment allocation until the last participant had completed follow-up. Patient characteristics were summarised as mean and standard deviation and/or median and interquartile range for each treatment arm and compared between arms using Wilcoxon rank-sum or Kruskal-Wallis tests.

Multivariable linear regression models were used to test for a difference in key outcomes between treatment arms while controlling for minimisation variables (centre, BMI (≤42 and >42 kg/m^2^), diabetes duration (≤5 and >5 years), insulin (yes, no)) and baseline measures for the analysed outcome. Outcomes investigated included differences in HbA1c at 6 months (primary outcome) and all other time points (3, 9, 12 months) as well as differences in body weight and % body weight at all time points (3, 6, 9, 12 months). Results are presented with 95% confidence intervals and p-values testing the impact of each independent variable on the outcome.

A multivariable logistic regression model following the same principle as the linear models was used to test for a difference in diabetes resolution between treatment arms. Diabetes resolution is defined as the patient achieving an HbA1c of <48 mmol/mol and off all diabetes medications.

## Results

**Fig. 2:**
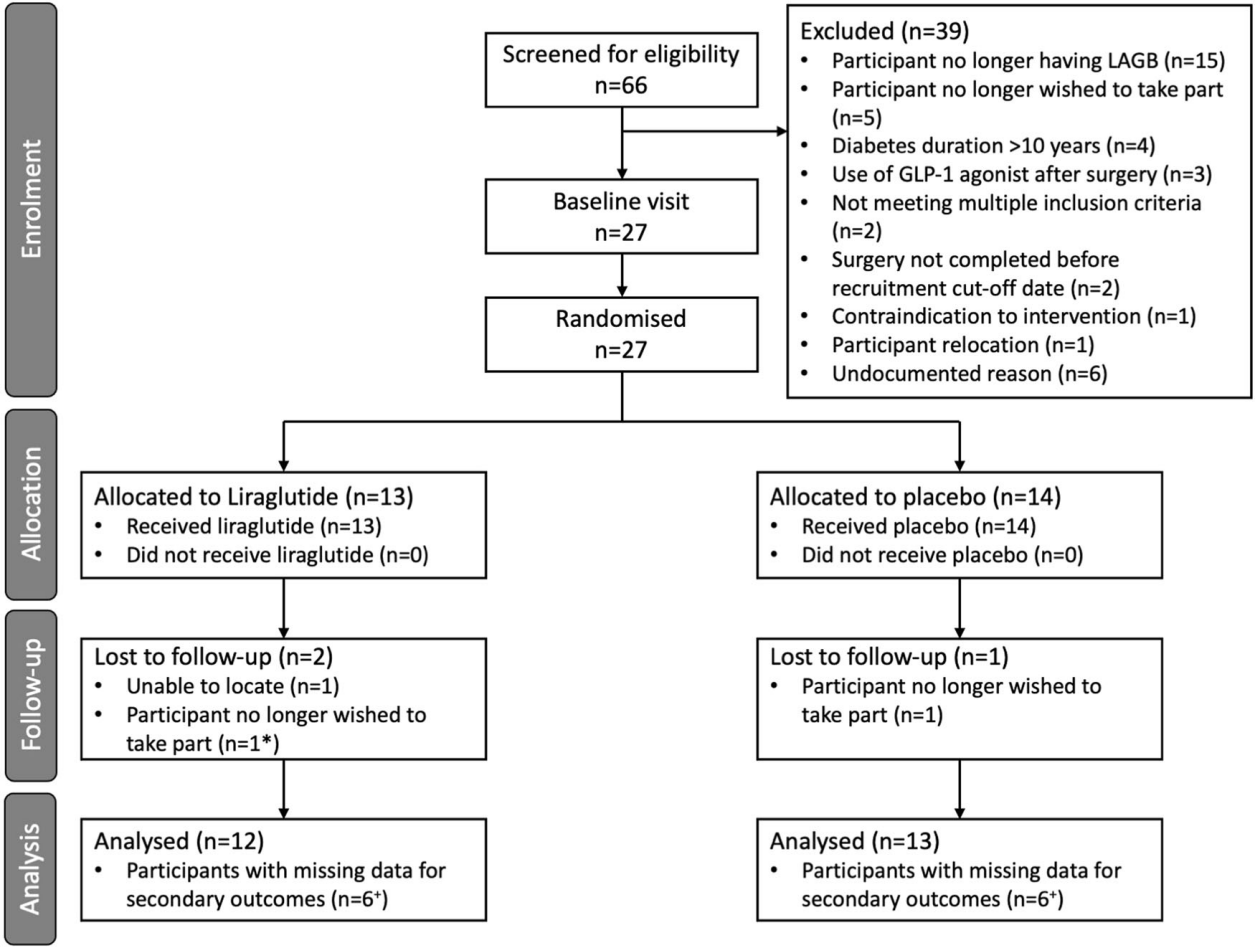
Trial CONSORT diagram. *Participant who no longer wished to take part from Liraglutide arm withdrew after 9 months of follow-up and was consequently included in the analysis of the primary outcome (and some secondary outcomes). ^+^Participants with missing secondary outcome data may be missing data for different outcomes or different timepoints.

Between 27/03/2018 and 25/03/2020, 66 participants were screened for eligibility. Of these participants 39 were screen failures and 27 participants were randomly assigned to subcutaneous liraglutide 1.8 mg once daily or placebo for 6 months. Twenty-seven of the target 58 participants were therefore randomised to this trial. Of the participants randomised two were lost to follow-up (*n* = 1 Liraglutide arm, *n* = 1 placebo arm) making a final sample of 25 participants for analysis (*n* = 12 liraglutide, *n* = 13 placebo) (Fig. 2). We identified no differences at randomisation between liraglutide and placebo, apart from a marginally higher systolic blood pressure in the placebo arm at randomisation (Table 1). T2DM was on average diagnosed 3 years before consent; for liraglutide this was 4 years (IQR:1–8) and for placebo this was 3 years (IQR:2–5). Supplementary Tables 2–4 provide participant medical history and concomitant medications.

**Table 1:** Demographics characteristics and physical examination at baseline.

	Total (*n* = 27)	Liraglutide (*n* = 13)	Placebo (*n* = 14)
Age at Randomisation	52.30 ± 8.38	53.48 ± 8.31	51.20 ± 8.59
Female Gender	21 (77.8%)	10 (76.9%)	11 (78.6%)
Ethnicity			
White	20 (74.1%)	8 (61.5%)	12 (85.7%)
Black	7 (25.9%)	5 (38.5%)	2 (14.3%)
Smoker?			
No	14 (51.9%)	6 (46.2%)	8 (57.1%)
No, but did in the past	10 (37.0%)	6 (46.2%)	4 (28.6%)
Yes, current smoker	3 (11.1%)	1 (7.7%)	2 (14.3%)
Drinks alcohol?			
No	10 (37.0%)	7 (53.8%)	3 (21.4%)
No, but did in the past	4 (14.8%)	3 (23.1%)	1 (7.1%)
Yes, current drinker	13 (48.1%)	3 (23.1%)	10 (71.4%)
Height (cm)	165.0 (160.0–174.0)	166.0 (160.0–174.0)	164.5 (160.0–174.0)
Weight (kg)	102.0 (91.7–123.3)	101.0 (94.3–128.5)	103.10 (87.1–116.0)
Body Mass Index	37.77 (33.20–43.66)	38.91 (35.18–46.11)	34.76 (32.11–42.87)
Neck circumference (cm)	39.0 (37.0–4400)	39.0 (37.0–45.0)	39.0 (37.0–42.0)
Hip circumference (cm)	118.0 (11200–139.0)	127.0 (112.0–146.0)	116.50 (112.0–132.0)
Waist circumference (cm)	116.0 (107.0–137.0)	121.0 (108.0–140.0)	113.0 (106.0–122.0)
Systolic Blood Pressure across 3 sittings (mmHg)	124.00 (114.00–127.00)	121.00 (111.33–124.67)	125.67 (123.67–130.00)
Diastolic Blood Pressure across 3 sittings (mmHg)	80.67 (73.67–86.00)	73.67 (71.67–85.00)	82.17 (79.00–86.00)
Pulse across 3 sittings (bpm)	70.67 (66.00–79.67)	76.67 (68.67–82.00)	70.17 (63.33–79.67)
Fat free mass (kg)	55.65 (49.70–68.30)	56.30 (49.70–67.50)	54.80 (49.50–72.90)
Missing	1	0	1
Fat mass (kg)	48.30 (38.10–61.00)	46.30 (42.80–61.00)	50.30 (36.00–58.10)
Missing	1	0	1
Fat percentage (%)	45.55 (40.50–48.80)	45.40 (43.20–47.50)	47.40 (40.50–48.80)
Missing	1	0	1
Year of Diagnosis (median)	2015 (2011–2017)	2015 (2010–2018)	2015 (2013–2017)
Time between T2DM diagnosis and consent (years, median)	3 (2–6)	4 (1–8)	3 (2–5)

There is no missing demographic data. Age is reported as Mean ± SD. The remaining variables are reported as *n*(%).

Physical Examination at baseline: Variables with missing data are indicated. All variables are reported as Median (Interquartile Range) due to a number of non-normally distributed variables.

*kg* kilogram, *cm* centimetre, *n* number.

### Primary outcome: HbA1c

#### HbA1c

**Fig. 3:**
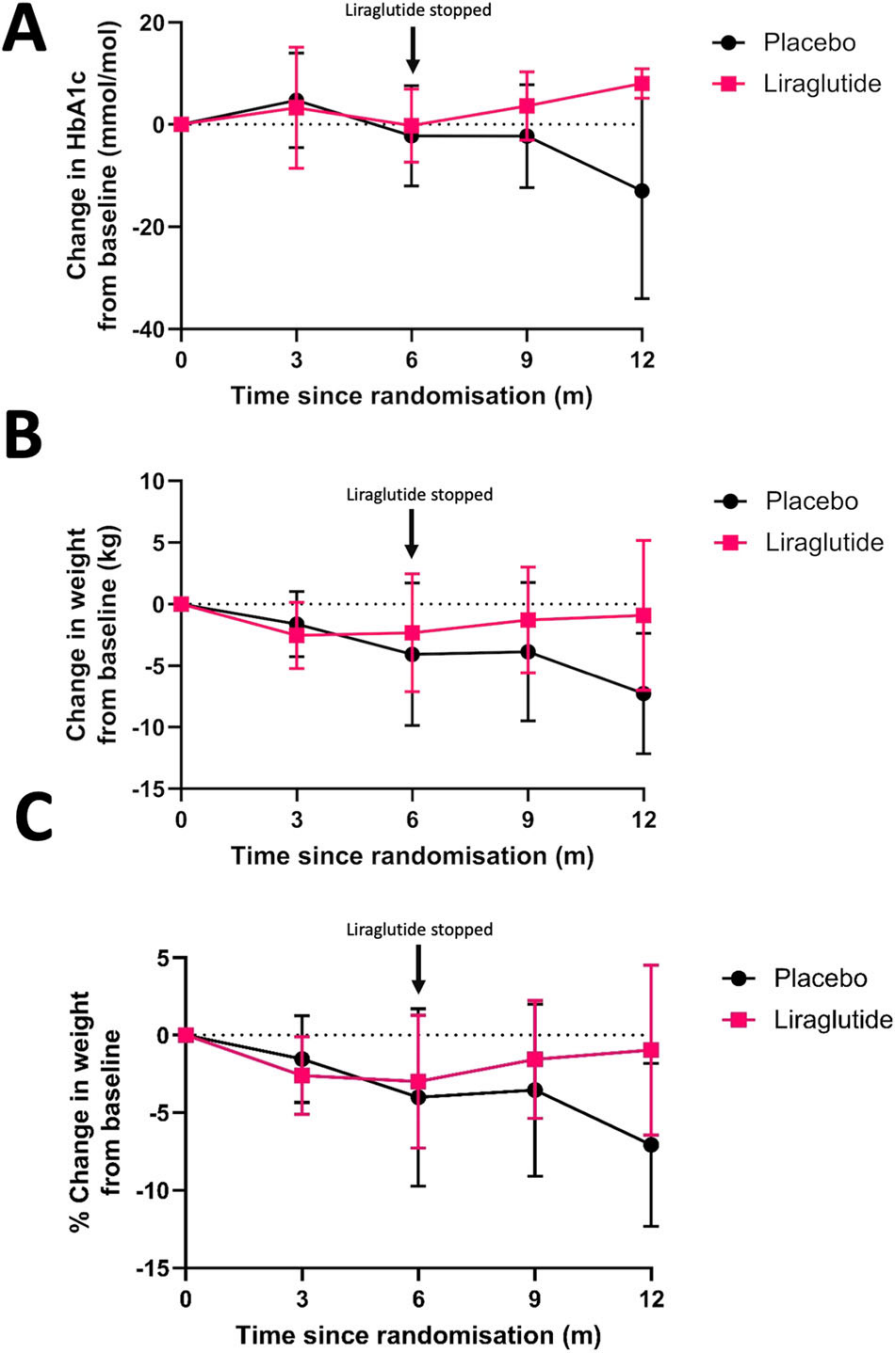
Graphs delineating body weight and HbA1c trend over 12 months. **A** – HbA1c, **B** – Body weight **C** – Percentage body weight change.

At screening, median HbA1c was 51 mmol/mol in the liraglutide (L) arm and 58 mmol/mol in the placebo (P) arm (*p* = 0.26). At randomisation, within 6 weeks of surgery, median HbA1c was 45.0 mmol/mol in the liraglutide arm and 55.0 mmol/mol in the placebo arm (*p* = 0.10), with no significant difference between groups. At 6 months, there was no significant change in median HbA1c from randomisation between groups (L:−0.5 mmol/mol, P −3.0 mmol/mol, *p* = 0.53, Table 2). At 12 months (and following cessation of liraglutide at 6 months), the liraglutide arm had significantly increased HbA1c compared to placebo (L: + 8.0 mmol/mol, *P*: −3.5 mmol/mol, p = 0.022, Table 2, Fig. 3, Supplementary Fig. 1). Pre-specified multivariate analysis after adjusting for screening HbA1c, BMI category (BMI > 42/≤42), duration of T2DM (>5 years/<5 years), insulin use and study site showed no significant difference in HbA1c at 6 months (0.2 mmol/mol, 95%CI −11.3, 11.6 mmol/mol, *p* = 0.98, Supplementary Table 5.2). Multivariate analysis of 12-month HbA1c reported that HbA1c significantly increased after cessation of liraglutide in the liraglutide group by ~10.9 mmol/mol when compared to placebo (10.9 mmol/mol, 95%CI: 1.1, 20.6 mmol/mol, *p* = 0.032, Supplementary Table 5.4). Both multivariate and univariate analysis of HbA1c at 3 and 9 months showed no significant differences between groups (Supplementary Table 5.1, Supplementary Table 5.3, Table 2) HbA1c values at 12 months were not collected for 9/27 participants (33.3%) (Fig. 3, Supplementary Fig. 1, Supplementary Fig. 5, Table 2, Supplementary Fig. 3.1, Supplementary Clinical Data 1).

**Table 2:** Difference in HbA1c and Body Weight at 6 months as well as at all other follow-up timepoints.

	HbA1c (mmol/mol)				Body weight (kg)			
	Total (*n* = 27)	Liraglutide (*n* = 13)	Placebo (*n* = 14)	*p* value	Total (*n* = 27)	Liraglutide (*n* = 13)	Placebo (*n* = 14)	*p* value
Screening	57.0 (48.0–65.0)	51.0 (46.0–63.0)	58.0 (54.0–65.0)	0.26	107.4 (97.8–127.5)	107.4 (101.0–128.5)	107.4 (96.8–123.5)	0.56
0 months	51.0 (43.0–63.0)	45.0 (42.0–59.0)	55.0 (48.0–65.0)	0.10	102.0 (91.7–123.3)	101.0 (94.3–128.5)	103.1 (87.1–116.0)	0.53
3 months								
Average	52.5 (43.0–80.5)	44.5 (41.0–75.0)	58.0 (49.0–86.0)	0.13	101.7 (87.3–122.2)	104.2 (88.8–135.6)	101.7 (84.9–118.1)	0.60
Change from 0 months	−0.5 (−4.0–7.5)	−4.0 (−6.0–7.0)	1.5 (−1.00–8.0)	0.31	−1.8 (−4.1–0.3)	−2.9 (−5.3–0.2)	−1.4 (−3.3–0.5)	0.45
% Change From Baseline					−1.72 (−4.56–0.34)	−2.34 (−5.12–0.01)	−1.25 (−3.58–0.38)	0.39
Missing	7	3	4		3	1	2	
6 months								
Average HbA1c	49.0 (42.0–63.0)	42.5 (39.0–69.5)	52.0 (44.0–61.0)	0.23	92.7 (86.6–121.2)	93.4 (86.6–140.3)	92.0 (82.1–115.0)	0.47
Change from 0 months	−3.0 (−11.0–3.0)	−0.5 (−9.5–6.5)	−3.0 (−11.0–3.0)	0.53	−2.8 (−7.7–2.0)	−2.6 (−7.6–1.9)	−2.9 (−10.0–3.8)	0.92
% Change from Baseline					−2.81 (−8.17–1.68)	−2.84 (−8.17–1.25)	−2.79 (−9.80–3.65)	0.92
Missing	2	1	1		5	2	3	
9 months								
Average	51.0 (44.0–67.0)	46.0 (42.0–67.0)	53.0 (45.0–70.0)	0.31	95.7 (82.4–118.5)	99.3 (86.5–135.0)	90.0 (80.9–113.8)	0.49
Change from 0 months	1.0 (−5.0–6.0)	3.0 (0.0–6.0)	−0.5 (−10.0–13.0)	0.27	−1.5 (−7.3–0.4)	−1.0 (−4.8–0.4)	−2.9 (−12.6–2.9)	0.45
% Change from Baseline					−1.29 (−7.74–0.53)	−0.89 (−3.89–0.53)	−2.61 (−12.94–3.33)	0.49
Missing	6	2	4		5	2	3	
12 months								
Average	54.5 (45.0–63.0)	56.0 (48.0–65.0)	54.5 (44.0–60.0)	0.59	88.9 (80.4–121.0)	106.7 (83.4–135.6)	88.0 (77.6–114.7)	0.16
Change from 0 months	5.5 (−7.0–8.0)	8.0 (6.50–10.0)	−3.5 (−13.0–6.0)	0.022	−4.4 (−9.4–1.7)	0.90 (−8.3–4.9)	−7.7 (−13.1–1.3)	0.067
% Change from Baseline					−3.14 (−9.48–1.68)	0.90 (−9.01–3.54)	−6.55 (−12.84–1.12)	0.11
Missing	9	5	4		6	3	3	

Variables with missing data are indicated. All variables are reported as Median (Interquartile Range) due to a number of non-normally distributed variables. Differences between treatment groups were examined using Wilcoxon rank-sum test. *N* = number.

#### Body weight

At screening, the groups had an equivalent body weight of 107.4 kg (Table 2). At randomisation, the median body weight was 101.0 kg in the liraglutide arm and 103.1 kg in the placebo. At 6 months, there was no significant change in median body weight between groups (L:−2.6 kg, P:−2.9 kg, *p* = 0.92, Table 2). At 12 months, the liraglutide group tended to increase body weight compared to the placebo (L:0.9 kg, P:−7.7 kg, *p* = 0.067, Table 2, Fig. 3, Supplementary Fig. 1). At 6 months multivariate analysis demonstrated no significant difference between groups (2.0 kg, 95%CI:−4.2, 8.1 kg, *p* = 0.50, Supplementary Table 6.2). The multivariate analysis reported that body weight at 12 months was 8.2 kg higher in the liraglutide group than placebo (8.23 kg, 95%CI: 1.6, 14.9 kg, *p* = 0.020, Supplementary Table 6.4). Multivariate and univariate analysis of body weight at 3 and 9 months demonstrated no significant difference between groups (Supplementary Table 6.1, Supplementary Table 6.3, Table 2). 22.2% (6/27) of 12-month body weight values were missing (Table 2, Fig. 3, Supplementary Fig. 1, Supplementary Fig. 5, Supplementary Fig. 3.2, Supplementary Clinical Data 1). At all time points there was no significant difference between groups in % body weight change. (Table 2, Supplementary Tables 7.1–7.4, Fig. 3) Body weight reduction at all timepoints was significantly correlated with HbA1c reduction (Supplementary Fig. 4).

Supplementary Results Description 1 provides explanations regarding participants cardiovascular risk factors, anthropometric parameters and quality of life throughout the trial.

### Diabetes remission and glycaemic control

On visual inspection of graphs depicting the distribution of T2DM duration between groups, (Supplementary Fig. 2), those in the liraglutide arm seemed to have longer duration of diabetes. However, objective statistical analysis of the duration of T2DM between groups did not show any significant difference (*p* = 0.23). There was no significant difference between groups in remission of diabetes at 6 months (L: 9.1%, P: 18.2%, *p* = 1.00) and 12 months (L:27.3%, C:25.0%, *p* = 1.00). (Supplementary Table 8). Measures of diabetes control (fasting glucose, fasting insulin, HOMA-IR) are presented in Supplementary Table 9. Multivariate analysis of fasting glucose showed that fasting glucose was lower in the placebo arm at 12 months (2.2 mmol/L 95%CI: 0.4, 4.1, *p* = 0.027) but not at 6 months (1.4 mmol/L, 95%CI −1.1, 3.9, *p* = 0.24) (Supplementary Tables 10.1 and 10.2). One hypoglycaemic event occurred in the placebo group. (Supplementary Clinical Sequelae 1).

### Concomitant medications

At randomisation, on average, individuals in both arms were treated with one glucose-lowering agent. There were 14 recorded medications for the liraglutide arm; all patients were treated with metformin (13/13), and one was additionally treated with insulin (Tresiba®/degludec insulin). For the placebo arm, there were 20 recorded medications, 100% (14/14) of patients were treated with metformin, 14.3% (2/14) were on dapagliflozin, 7.1% (1/14) were treated with either gliclazide, Humulin I®, Insulutard® or Victoza®. During follow-up, 6 new glucose-lowering medications were used in the liraglutide group and 3 new glucose-lowering medications in the placebo group. For the liraglutide group participants were started on: 2 metformin, 2 empagliflozin, 1 canagliflozin, 1 semaglutide. For the placebo arm participants were started on: 1 metformin, 2 dapagliflozin (Supplementary Table 4).

### Adverse events

Forty-two adverse events (AE) occurred during the trial: 32 were in the liraglutide arm, and 10 were in the placebo arm. There were three serious adverse events (SAEs) during the trial: 2 in the liraglutide arm and 1 in the placebo arm. The most serious occurred in the placebo arm: cholecystitis requiring cholecystectomy. Investigational medicinal product was stopped for 5 participants in the liraglutide arm due to adverse events (5/13). Twenty-seven gastro-intestinal symptom-related AEs occurred: 20 in the liraglutide arm and 7 in the placebo arm. Gastric Band-related side effects also occurred more commonly in the liraglutide arm (L: 15 band-related AEs, P: 5 band-related AEs). 7 adverse events were ongoing at the end of the study, all in the liraglutide arm. Gastrointestinal side-effects occurred more frequently in the liraglutide arm, the most frequent being vomiting (*n* = 6, 46.2%) (Table 3).

**Table 3:** Adverse events.

	Total (*n* = 27)	Liraglutide (*n* = 13)	Placebo (*n* = 14)	*p* value
Patient experiences				
Average number of AEs	3.0 (2.0–4.0)	3.5 (2.0–5.5)	2.5 (1.5–3.5)	0.39
Patient with surgery-related AEs	2 (16.7%)	2 (25.0%)	0 (0.0%)	0.52
Patient with gastric band related AEs	8 (66.7%)	6 (75.0%)	2 (50.0%)	0.55
Across patients				
Number of AEs	42	32	10	n/a
Number of SAEs	3	2	1	n/a
Severity of AE				
Mild	27	21	6	
Moderate	7	4	3	
Severe	8	7	1	
IMP Cause of AE				
Not related	16	12	4	
Unlikely	2	0	2	
Possible	16	14	2	
Probable	6	5	1	
Dose stopped due to AE	5	5	0	n/a
Con Med commenced after AE	10	7	3	n/a
Number of surgery-related AEs	5	5	0	n/a
Number of gastric band-related AEs	20	15	5	n/a
Number of AEs with GI symptoms	27	20	7	n/a
Average time (days) between randomisation and AE	41.0 (1.0–201.0)	6.0 (−16.0–45.0)	228.5 (154.0–230.0)	n/a
Average duration of AEs (days)	11.0 (3.0–43.0)	11.0 (2.0–35.0)	21.5 (7.0–43.0)	n/a
Number of AEs ongoing at study end	7	7	0	n/a
Outcome of AE				
Recovered	33	25	8	
Resolved with sequelae	1	0	1	
No recovery	5	4	1	
Further Breakdown of Adverse Events				
Number of patients with adverse events	12 (44.4%)	8 (61.5%)	4 (28.6%)	
Gastrointestinal Events				
Nausea	6 (22.2%)	6 (46.2%)	0	
Diarrhoea	3 (11.1%)	1 (7.7%)	2 (14.3%)	
Constipation	1 (3.7%)	1 (7.7%)	0	
Vomiting	3 (11.1%)	2 (15.4%)	1 (7.1%)	
Abdominal discomfort	3 (11.1%)	2 (15.4%)	1 (7.1%)	
Gastro-oesophageal reflux	2 (7.4%)	0	2 (14.3%)	
Loss of appetite	2 (7.4%)	2 (15.4%)	0	
Bloating	1 (3.7%)	1 (7.7%)	0	
Dry mouth	1 (3.7%)	1 (7.7%)	0	
General Events				
Cramps (not GI)	1 (3.7%)	1 (7.7%)	0	
Irritable legs	1 (3.7%)	1 (7.7%)	0	
Sweating	1 (3.7%)	1 (7.7%)	0	
Itching	1 (3.7%)	1 (7.7%)	0	
Cough	1 (3.7%)	0	1 (7.1%)	
Numbness/cold to extremities	2 (7.4%)	2 (15.4%)	0	
Pain				
Chest pain	1 (3.7%)	1 (7.7%)	0	
Migraine	1 (3.7%)	1 (7.7%)	0	
Sciatic Pain	1 (3.7%)	1 (7.7%)	0	
Pain of right side flank	1 (3.7%)	0	1 (7.1%)	
Vascular events				
Dizziness	11 (3.7%)	1 (7.7%)	0	
Infections				
Thrush	2 (7.4%)	1 (7.7%)	1 (7.1%)	
Urinary Tract Infection	1 (3.7%)	1 (7.7%)	0	
Serious adverse events				
Cholecystitis	1 (3.7%)	0	1 (7.1%)	
Further Details: Underwent cholecystectomy				
Pain of left upper quadrant	1 (3.7%)	1 (7.7%)	0	
Further Details: Started analgesia + anti-emetics. AE was related to gastric band.				
Abdominal Pain	1 (3.7%)	1 (7.7%)	0	
Further Details: IMP stopped. AE potentially related to gastric band or IMP.				

Average number of adverse events (AEs) per patient as well as average time to AE and duration of AE across patients is reported as median (interquartile range). The number of patients with surgery-related and gastric band-related AEs is reported as *n*(%). Total number of AEs with differing severity categories, outcomes, causes etc. across AEs is reported as the number of recorded entries. Difference between groups in average number of AEs per patient is examined using a Wilcoxon rank-sum test. Differences between treatment groups in the number of patients with surgery-related and gastric band-related AEs is examined using Fisher’s exact test. n/a=Differences between treatment groups were not examined.

### Band adjustments

Four participants (two in each treatment arm) had no reported band adjustments. On average participants had 3 band adjustments during the trial, this number did not significantly differ between groups (L:3.0, P:3.0, *p* = 0.39). Participants had two band adjustments over first 6 months and one band adjustment over the 6 month follow-up period (Supplementary Table 16). The number of band adjustments significantly differed between sites (*p* = 0.016, Kruskal-Wallis test). However, this difference was not specific to individual treatment arms. GSTT (*n* = 16) tended to perform 3.0 (0.5–3.5) adjustments per participant, whilst North Bristol (*n* = 7) performed more adjustments, 4.0 (3.0–6.0). BHH performed two adjustments for each of their three participants.

## Discussion

The GLIDE trial aimed to assess the metabolic impact of the addition of a GLP-1 receptor agonist in the form of liraglutide 1.8 mg once daily following a LAGB in patients with T2DM. We hypothesised that the addition of a GLP-1 receptor agonist would increase the efficacy of LAGB compared to LAGB alone. To our knowledge, this is the first randomised, double-blind, placebo-controlled trial investigating the impact of GLP-1 receptor agonist treatment following LAGB insertion.

Our findings did not reveal significant differences in either HbA1c or weight at 6 months between the LAGB and liraglutide arm compared to LAGB and placebo. However, after cessation of IMP at 6 months, those in the liraglutide arm experienced both a statistically significant rise in HbA1c and weight that was not seen in the placebo arm. These results may be reflective of a suppressant effect of liraglutide on weight and HbA1c in the treatment arm. However, the trial’s target sample size was 58 in total and was therefore underpowered to detect a significant difference in the primary and secondary endpoints at all time points.

Remission of T2DM in both the liraglutide and placebo arms was similar, with three patients in both groups achieving full remission of T2DM at 12 months as defined by HbA1c < 48 mmol/mol and stopping all diabetes medication. Weight loss of around 10–15% leads to remission of T2DM in patients with early T2DM [32, 33]. In the GLIDE trial, patients with a duration of T2DM greater than ten years were excluded. Observational studies and clinical practice suggest that these patients would benefit more from an LSG or RYBG intervention than LAGB, although there are currently no published randomised clinical trials showing the superiority of one procedure in terms of long-term glycaemic control and remission of T2DM [10, 34]. Of note, the By-Band-Sleeve study is a pragmatic clinical trial assessing LAGB, RYGB, and LSG. This trial is ongoing and the results will provide robust evidence of the impact of various bariatric surgical procedures on long-term glycaemic control and T2DM remission [35, 36].

Importantly, whilst there was no significant difference between groups in diabetes duration at screening, graphs showed that seven participants in liraglutide arm had duration ≥5 years, but only four participants in placebo had duration ≥5 years. In addition, two participants in the liraglutide and seven in the placebo arm had diabetes duration ≤2 years duration. Diabetes duration affects chance of diabetes remission following weight loss [37]. For instance, an observational study showed complete remission of T2DM inversely correlated with duration of diabetes, with remission rates highest in those with a more recent diagnosis [34]. Duration of diabetes may, therefore, partly explain the glycaemic findings reported in our study.

There were no other significant metabolic differences between the two arms at 6 and 12 months, except for an improvement in diastolic blood pressure in the placebo arm. This is likely to have been driven by the additional weight loss in the placebo arm compared to the relative weight gain after cessation of GLP-1 therapy in the liraglutide arm. No significant differences were observed between the two groups in terms of quality-of-life scores, indicating that additional liraglutide therapy is safe and not detrimental to quality of life.

This randomised controlled trial had a target sample size of 58 (29 per group), however only 27 participants were randomised to the trial. This trial was therefore significantly underpowered to detect a difference between groups in the primary and secondary endpoints. This principally relates to the change in practice over time whereby the LAGB is now less popular, with RYGB and LSG being the preferred procedures. However, this data does provide important pilot data regarding adjuvant GLP-1 receptor agonist therapy following bariatric surgery.

Our study showed no differences in HbA1c or weight at 6 months between the two arms. However given the treatment duration of only 6 months, small sample size and the use of relatively less powerful adjunctive therapy further evaluation may be warranted post-metabolic surgery. A Study in rodents, although without diabetes, revealed that the administration of a GLP-1 agonist enhanced the effect of gastric banding [38]. Moreover, Miras et al., in the GRAVITAS randomised clinical trial (*n* = 80), tested 26 weeks of adjuvant liraglutide 1.8 mg compared to placebo in participants with persistent or recurrent T2DM post-bariatric surgery (RYGB or LSG). The investigators reported significant improvements in HbA1c versus placebo with adjuvant liraglutide [39].

Additional observational and clinical studies have evaluated liraglutide post-bariatric surgery [40–44]. In a longitudinal study (*n* = 117) of post-bariatric surgery liraglutide at the higher dose of 3.0 mg, patients had significant weight loss irrespective of initial surgery (RYGB −7.1 ± 8.7 kg, LAGB: −6.0 ± 7.2 kg, LSG: −4.5 ± 3.5) [41]. Similarly, a prospective study reported that ≥16 weeks of liraglutide 3.0 mg therapy led to 6.4% median weight loss, with no significant difference between groups in magnitude of weight loss between non-surgical and bariatric surgical patients [42]. In a case-matched study of adjuvant liraglutide following retrieval of the intra-gastric balloon, there was significantly less weight regain in the liraglutide group than the placebo (−1.2 kg ± 0.9, −0.7 ± 1.0, *p* = 0.010). Notably, the effect size was very marginal, akin to the negative findings reported in our study [43]. For endoscopic procedures, a retrospective investigation reported endoscopic sleeve gastrectomy (ESG) with adjuvant liraglutide led to significantly greater 7 month total body weight reduction (24.7% ± 2.1, 20.5% ± 1.7, *p* < 0.001) than the ESG alone group [45]. A RCT (*n* = 23, liraglutide between 6 weeks and 6 months post-op) randomised participants to 3.0 mg liraglutide or placebo after laparoscopic sleeve gastrectomy. The liraglutide arm had significantly greater %-Estimated weight loss (liraglutide: 58.7% %EWL, placebo: 44.5%, *p* = 0.043) and resolution of dysglycaemia (liraglutide: 100% resolution, Placebo: 50%) than placebo. This trial used a higher liraglutide dose [44]. Overall our data contradict these previous findings, with the reasons underpinning likely being related to the timing of GLP-1 initiation, dosing of liraglutide, duration of treatment and the agent choice. It remains to be determined through randomised controlled trials whether novel agents like semaglutide or tirzepatide may be of utility post-metabolic surgery.

The number of glucose-lowering therapies varied between trial groups during follow-up. In the liraglutide arm six patients commenced new medications, whereas only three patients in the placebo arm did. This may be due to liraglutide being stopped at 6 months following randomisation, and consequently glycaemic control worsened. In addition, as per the trial protocol, we stopped metformin if HbA1c < 48 mmol/mol. In the intervention arm the HbA1c at 6 months was on average 42.5 mmol/mol, whereas in placebo was 52.0 mmol/mol. Finally, liraglutide was stopped in five instances due to AE, this could have contributed towards sub-optimal glycaemic response.

The study intervention was safe, with no statistical differences reported in total adverse events between the placebo and liraglutide arms. There was a numerically higher number of gastrointestinal AEs with liraglutide, in line with previous experience with GLP-1 therapy.. There was a numerically higher number of gastric-band-related AEs in the liraglutide arm vs placebo, although unlikely to be related to GLP-1 therapy. The number of band adjustments was similar in both groups, with an average of 3 band adjustments over 12 months. This number is generally lower than seen in routine clinical practice over 12 months and is likely to be related to a reduction in face-face appointments and limited band adjustments during the Covid-19 pandemic. This may also explain the suboptimal response to gastric band intervention in both groups in terms of body weight and glycaemic response. Importantly, in terms of serious adverse events there was one in the placebo arm (cholecystitis) and two in liraglutide arm (left upper quadrant pain and abdominal pain). The cholecystitis required cholecystectomy and hospital admission. The left upper quadrant abdominal pain required analgesia and anti-emetics, and the abdominal pain required cessation of IMP. Overall, this shows the safety of liraglutide in combination with LAGB.

### Strengths and limitations

The GLIDE clinical trial has several strengths. It used a randomised, double-blind, placebo-controlled design which increased the validity of the results. The study mirrors routine clinical care to reduce the patient burden taking part in the research. Finally the study provides data to power further randomised controlled trials.

However, the trial has several limitations. Importantly, the study did not achieve its estimated sample size, with several reasons accounting for this. Firstly, we noted a change in clinical practice over the time of the trial, with a general preference for a LSG or RYGB procedure compared to LAGB, especially in participants with T2DM. Secondly, inclusion criteria limited the duration of T2DM for participants to <10 years, excluding many of our patients from the trial who often present with a long duration of T2DM. Thirdly, the COVID-19 pandemic significantly impacted the study, with the majority of participants either randomised just before the pandemic or reaching the critical 6 month and 12-month trial milestones during the pandemic. This resulted in missing critical primary and secondary outcome data for some participants, including weight and HbA1c data, and restricted face-to-face appointments for band adjustment. This may partly underpin the sub-optimal metabolic and weight outcomes in both groups. A further limitation was the study’s relatively short duration (26 weeks) with liraglutide 1.8 mg. The GLIDE trial showed the worsening effect of HbA1c and weight after cessation of therapy, and it would have been of interest to determine the longer-term impact of GLP-1 therapy beyond 6 months. As per its license for the treatment of T2DM, participants were given liraglutide at a maximum dose of 1.8 mg once daily. Evidence has shown more significant weight loss with liraglutide 3 mg; at 6 months, some patients may have not yet achieved their nadir weight [9]. Furthermore recent retrospective evidence suggests that 1.0 mg semaglutide is superior to 3.0 mg liraglutide in management of weight regain following metabolic surgery [46]. The study is also limited by only following up patients to 1 year, excess weight loss following LAGB may peak at 2 years follow-up [47]. The final limitation is the low number of band adjustments performed during the first 6 months which is likely to explain the much lower than expected weight loss at 6 months and consequently the attenuated reduction in HbA1c.

## Conclusions

In conclusion, our pilot randomised controlled trial showed that the addition of the GLP-1 agonist receptor liraglutide after LABG did not significantly improve HbA1c or weight compared to placebo at 6 months. Importantly this trial was underpowered to detect a significant difference between groups in the primary and secondary endpoints. Results are limited by the relatively small sample size and the sub-optimal number of band-adjustments which is, in part, related to the COVID pandemic and may underpin the sub-optimal weight and glycaemic responses in both groups. Future larger randomised controlled trials of longer duration, more intensive LAGB follow-up and with more effective agents (i.e. semaglutide, tirzepatide) are required to confirm whether adjunctive GLP-1 agonist therapy and other gut hormone therapies are beneficial post-metabolic surgery in patients with T2DM.

### Supplementary information

Supplementary Material

## Data Availability

Data for the trial are available upon reasonable request to the corresponding author.

## References

[1] World Health Organization. Obesity and Overweight: WHO n.d. https://www.who.int/news-room/fact-sheets/detail/obesity-and-overweight (accessed 21 Mar 2023).

[2] Chin SH, Kahathuduwa CN, Binks M (2016). Physical activity and obesity: what we know and what we need to know*. Obes Rev.

[3] Carbone S, Del Buono MG, Ozemek C, Lavie CJ (2019). Obesity, risk of diabetes and role of physical activity, exercise training and cardiorespiratory fitness. Prog Cardiovasc Dis.

[4] Agha M, Agha R (2017). The rising prevalence of obesity. Int J Surg Oncol.

[5] Ayton A, Ibrahim A (2019). Obesity is a public health emergency. BMJ.

[6] Wild S, Roglic G, Green A, Sicree R, King H (2004). Global prevalence of diabetes: estimates for the year 2000 and projections for 2030. Diabetes Care.

[7] Dobbie LJ, Tahrani A, Alam U, James J, Wilding J, Cuthbertson DJ. Exercise in obesity—the role of technology in health services: can this approach work? *Curr Obes Rep.* 2021. 10.1007/s13679-021-00461-x 10.1007/s13679-021-00461-x PMC8597870 34791611

[8] Wilding JPH, Batterham RL, Calanna S, Davies M, Van Gaal LF, Lingvay I (2021). Once-weekly semaglutide in adults with overweight or obesity. N Engl J Med.

[9] Pi-Sunyer X, Astrup A, Fujioka K, Greenway F, Halpern A, Krempf M (2015). A randomized, controlled trial of 3.0 mg of liraglutide in weight management. N Engl J Med.

[10] Puzziferri N, Roshek TB, Mayo HG, Gallagher R, Belle SH, Livingston EH (2014). Long-term follow-up after bariatric surgery: a systematic review. JAMA.

[11] Jensen AB, Renström F, Aczél S, Folie P, Biraima-Steinemann M, Beuschlein F, et al. Efficacy of the glucagon-like peptide-1 receptor agonists liraglutide and semaglutide for the treatment of weight regain after bariatric surgery: a retrospective observational study. *Obes Surg.* 2023. 10.1007/s11695-023-06484-8. 10.1007/s11695-023-06484-8 PMC9918402 36765019

[12] National Institute for Health and Care Excellence. Obesity: clinical assessment and management (QS127). *Natl Inst Heal Care Excell* 2016:44.

[13] Taheri S. Bariatric surgery: what’s the score? 2011;11:1–3. 10.1177/1474651410398816.

[14] Sjöström L, Narbro K, Sjöström CD, Karason K, Larsson B, Wedel H (2007). Effects of bariatric surgery on mortality in Swedish obese subjects. N Engl J Med.

[15] Adams TD, Gress RE, Smith SC, Halverson RC, Simper SC, Rosamond WD (2007). Long-term mortality after gastric bypass surgery. N Engl J Med.

[16] Buchwald H, Avidor Y, Braunwald E, Jensen MD, Pories W, Fahrbach K (2004). Bariatric surgery: a systematic review and meta-analysis. JAMA.

[17] Holter MM, Dutia R, Stano SM, Prigeon RL, Homel P, McGinty JJ (2017). Glucose metabolism after gastric banding and gastric bypass in individuals with type 2 diabetes: Weight loss effect. Diabetes Care.

[18] Ding L, Fan Y, Li H, Zhang Y, Qi D, Tang S, et al. Comparative effectiveness of bariatric surgeries in patients with obesity and type 2 diabetes mellitus: a network meta-analysis of randomized controlled trials. *Obes Rev.* 2020;21. 10.1111/obr.13030. 10.1111/obr.13030 PMC7379237 32286011

[19] Robertson AGN, Wiggins T, Robertson FP, Huppler L, Doleman B, Harrison EM (2021). Perioperative mortality in bariatric surgery: meta-analysis. Br J Surg.

[20] Tsouristakis AI, Febres G, McMahon DJ, Tchang B, Conwell IM, Tsang AJ (2019). Long-term modulation of appetitive hormones and sweet cravings after adjustable gastric banding and Roux-en-Y gastric bypass. Obes Surg.

[21] Bunt JC, Blackstone R, Thearle MS, Vinales KL, Votruba S, Krakoff J (2017). Changes in glycemia, insulin and gut hormone responses to a slowly ingested solid low-carbohydrate mixed meal after laparoscopic gastric bypass or band surgery. Int J Obes.

[22] Knudsen LB, Nielsen PF, Huusfeldt PO, Johansen NL, Madsen K, Pedersen FZ (2000). Potent derivatives of glucagon-like peptide-1 with pharmacokinetic properties suitable for once daily administration. J Med Chem.

[23] Elbrond B, Jakobsen G, Larsen S, Agerso H, Jensen LB, Rolan P (2002). Pharmacokinetics, pharmacodynamics, safety, and tolerability of a single-dose of NN2211, a long-acting glucagon-like peptide 1 derivative, in healthy male subjects. Diabetes Care.

[24] Blonde L, Russell-Jones D (2009). The safety and efficacy of liraglutide with or without oral antidiabetic drug therapy in type 2 diabetes: an overview of the LEAD 1-5 studies. Diabetes, Obes Metab.

[25] Buse JB, Garber A, Rosenstock J, Schmidt WE, Brett JH, Videbæk N (2011). Liraglutide treatment is associated with a low frequency and magnitude of antibody formation with no apparent impact on glycemic response or increased frequency of adverse events: results from the Liraglutide Effect and Action in Diabetes (LEAD) trials. J Clin Endocrinol Metab.

[26] Zinman B, Gerich J, Buse JB, Lewin A, Schwartz S, Raskin P (2009). Efficacy and safety of the human glucagon-like peptide-1 analog liraglutide in combination with metformin and thiazolidinedione in patients with type 2 diabetes (LEAD-4 Met+TZD). Diabetes Care.

[27] Russell-Jones D, Vaag A, Schmitz O, Sethi BK, Lalic N, Antic S (2009). Liraglutide vs insulin glargine and placebo in combination with metformin and sulfonylurea therapy in type 2 diabetes mellitus (LEAD-5 met+SU): a randomised controlled trial. Diabetologia.

[28] Garber A, Henry R, Ratner R, Garcia-Hernandez PA, Rodriguez-Pattzi H, Olvera-Alvarez I (2009). Liraglutide versus glimepiride monotherapy for type 2 diabetes (LEAD-3 Mono): a randomised, 52-week, phase III, double-blind, parallel-treatment trial. Lancet.

[29] Marre M, Shaw J, Brändle M, Bebakar WMW, Kamaruddin NA, Strand J (2009). Liraglutide, a once-daily human GLP-1 analogue, added to a sulphonylurea over 26 weeks produces greater improvements in glycaemic and weight control compared with adding rosiglitazone or placebo in subjects with Type 2 diabetes (LEAD-1 SU). Diabet Med.

[30] Nauck M, Frid A, Hermansen K, Shah NS, Tankova T, Mitha IH (2009). Efficacy and safety comparison of liraglutide, glimepiride, and placebo, all in combination with metformin, in type 2 diabetes: the LEAD (liraglutide effect and action in diabetes)-2 study. Diabetes Care.

[31] Buse JB, Rosenstock J, Sesti G, Schmidt WE, Montanya E, Brett JH (2009). Liraglutide once a day versus exenatide twice a day for type 2 diabetes: a 26-week randomised, parallel-group, multinational, open-label trial (LEAD-6). Lancet.

[32] Lean ME, Leslie WS, Barnes AC, Brosnahan N, Thom G, McCombie L (2018). Primary care-led weight management for remission of type 2 diabetes (DiRECT): an open-label, cluster-randomised trial. Lancet.

[33] Lean MEJ, Leslie WS, Barnes AC, Brosnahan N, Thom G, McCombie L (2019). Durability of a primary care-led weight-management intervention for remission of type 2 diabetes: 2-year results of the DiRECT open-label, cluster-randomised trial. Lancet Diabetes Endocrinol.

[34] Jans A, Näslund I, Ottosson J, Szabo E, Näslund E, Stenberg E. Duration of type 2 diabetes and remission rates after bariatric surgery in Sweden 2007-2015: a registry-based cohort study. *PLoS Med.* 2019;16. 10.1371/JOURNAL.PMED.1002985 10.1371/journal.pmed.1002985 PMC6867594 31747392

[35] Rogers CA, Welbourn R, Byrne J, Donovan JL, Reeves BC, Wordsworth S, et al. The By-Band study: gastric bypass or adjustable gastric band surgery to treat morbid obesity: study protocol for a multi-centre randomised controlled trial with an internal pilot phase. *Trials.* 2014;15. 10.1186/1745-6215-15-53 10.1186/1745-6215-15-53 PMC3942168 24517309

[36] Rogers CA, Reeves BC, Byrne J, Donovan JL, Mazza G, Paramasivan S (2017). Adaptation of the By-Band randomized clinical trial to By-Band-Sleeve to include a new intervention and maintain relevance of the study to practice. Br J Surg.

[37] Arterburn DE, Bogart A, Sherwood NE, Sidney S, Coleman KJ, Haneuse S (2013). A multisite study of long-term remission and relapse of type 2 diabetes mellitus following gastric bypass. Obes Surg.

[38] Habegger KM, Kirchner H, Yi CX, Heppner KM, Sweeney D, Ottaway N (2013). GLP-1R agonism enhances adjustable gastric banding in diet-induced obese rats. Diabetes.

[39] Miras AD, Pérez-Pevida B, Aldhwayan M, Kamocka A, McGlone ER, Al-Najim W (2019). Adjunctive liraglutide treatment in patients with persistent or recurrent type 2 diabetes after metabolic surgery (GRAVITAS): a randomised, double-blind, placebo-controlled trial. Lancet Diabetes Endocrinol.

[40] Rye P, Modi R, Cawsey S, Sharma AM (2018). Efficacy of high-dose liraglutide as an adjunct for weight loss in patients with prior bariatric surgery. Obes Surg.

[41] Wharton S, Kuk JL, Luszczynski M, Kamran E, Christensen RAG (2019). Liraglutide 3.0 mg for the management of insufficient weight loss or excessive weight regain post-bariatric surgery. Clin Obes.

[42] Suliman M, Buckley A, Al Tikriti A, Tan T, le Roux CW, Lessan N (2019). Routine clinical use of liraglutide 3 mg for the treatment of obesity: outcomes in non-surgical and bariatric surgery patients. Diabetes Obes Metab.

[43] Badurdeen D, Hoff AC, Barrichello S, Hedjoudje A, Itani MI, Farha J (2021). Efficacy of liraglutide to prevent weight regain after retrieval of an adjustable intra-gastric balloon-a case-matched study. Obes Surg.

[44] Thakur U, Bhansali A, Gupta R, Rastogi A (2021). Liraglutide augments weight loss after laparoscopic sleeve gastrectomy: a randomised, double-blind, placebo-control study. Obes Surg.

[45] Badurdeen D, Hoff AC, Hedjoudje A, Adam A, Itani MI, Farha J (2021). Endoscopic sleeve gastroplasty plus liraglutide versus endoscopic sleeve gastroplasty alone for weight loss. Gastrointest Endosc.

[46] Murvelashvili N, Xie L, Schellinger JN, Mathew MS, Marroquin EM, Lingvay I, et al. Effectiveness of semaglutide versus liraglutide for treating post-metabolic and bariatric surgery weight recurrence. *Obesity* 2023. 10.1002/oby.23736 10.1002/oby.23736 PMC12398307 36998152

[47] O’Brien PE, Hindle A, Brennan L, Skinner S, Burton P, Smith A (2019). Long-term outcomes after bariatric surgery: a systematic review and meta-analysis of weight loss at 10 or more years for all bariatric procedures and a single-centre review of 20-year outcomes after adjustable gastric banding. Obes Surg.

