# Supplementary material for: Laparoscopic adjustable gastric banding with liraglutide in adults with obesity and type 2 diabetes (GLIDE): a pilot randomised placebo controlled trial
Source: Int J Obes (Lond). 2023 Sep 11;47(11):1132–42. doi: 10.1038/s41366-023-01368-4 (PMC10599987; doi:10.1038/s41366-023-01368-4)
Supplement: Supplementary file 1 — Supplementary Material [file 41366_2023_1368_MOESM1_ESM.docx]

**Laparoscopic Adjustable Gastric Banding with Liraglutide in Adults with Obesity and Type 2 Diabetes (GLIDE): A Pilot Randomised Placebo Controlled Trial**

***Supplementary Material***

# Claudia Coelho^1^, Laurence J Dobbie^1^, James Crane^2^, Abdel Douiri^3^, Annastazia E Learoyd^3^, Olanike Okolo^1^, Sypros Panagiotopoulos^4^, Dimitri J Pournaras^5^, Sasindran Ramar^4^, Francesco Rubino^6^, Rishi Singhal^7^, Carel W le Roux^8^, Shahrad Taheri^9^, Barbara Mcgowan^1^

# ^1^Department of Diabetes and Endocrinology, Guy's and St Thomas' NHS Foundation Trust, London, UK ^2^Department of Endocrinology, King's College Hospital NHS Foundation Trust, London, UK ^3^School of Population Health and Environmental Sciences, King’s College London, London, UK ^4^Department of Minimal Access Surgery, King's College Hospital NHS Foundation Trust, London, UK ^5^Department of Upper GI and Bariatric/Metabolic Surgery, North Bristol NHS Trust, Southmead Hospital, Bristol, UK ^6^Department of Diabetes, School of Life Course Sciences, King's College London, London, U.K. ^7^Upper GI Unit at Heart of England, NHS Foundation Trust, Birmingham ^8^Diabetes Complications Research Centre, University College Dublin, Dublin, Ireland ^9^Department of Medicine, Weill Cornell Medicine Qatar, Doha, Qatar

# Corresponding Author

# Prof Barbara McGowan

# Email: Barbara.McGowan@gstt.nhs.uk

**Contents**

- Page 3: Supplementary Figure 1: Change in HbA1c & Body Weight Compared to Randomisation
- Page 4: Supplementary Figure 2: Descriptive analysis of length of T2DM
- Page 5: Supplementary Figure 3: Patient Trajectories for HbA1c and Body Weight
- Page 6: Supplementary Figure 4: Scatter Plot of Relationship between Change in HbA1c (y axis) and change in weight (x-axis)
- Page 7: Supplementary Figure 5: Boxplots of HbA1c & Body Weight Change over 12 months
- Page 8: Supplementary Table 1: Inclusion and Exclusion Criteria
- Page 9: Supplementary Table 2: Medical History
- Page 10: Supplementary Table 3: Concomitant medication at date of randomisation and additional medication during follow-up
- Page 11: Supplementary Table 4: Concomitant glucose control medication at date of randomisation and additional medication during follow-up
- Page 12: Supplementary Table 5: Multivariate Analysis of HbA1c Across all time points
- Page 13: Supplementary Table 6: Multivariate Analysis of Body Weight Change at All Time Points
- Page 14: Supplementary Table 7: Multivariate Analysis of Percentage Body Weight Change at All Timepoints
- Page 15: Supplementary Table 8: Remission of diabetes
- Page 16-17: Supplementary Table 9: Measures of Diabetes
- Page 18: Supplementary Table 10: Multivariate Analysis of Fasting Glucose
- Page 19-20: Supplementary Table 11: Anthropometric measures and Body Composition across follow-up
- Page 21-23: Supplementary Table 12: Cardiovascular Disease Risk Factors across follow-up
- Page 24-25: Supplementary Table 13: Impact of Weight on Quality of Life (IWQoL) measures across follow-up
- Page 26: Supplementary Table 14: EQ-5D-5L measures across follow-up
- Page 27: Supplementary Table 15: Hospital Anxiety and Depression Scale (HADS) across follow-up
- Page 28: Supplementary Table 16: Gastric Band Adjustments
- Page 29: Supplementary Table 17: Site Specific Analysis for HbA1c and Body Weight
- Page 30: Supplementary Clinical Data 1: Missing HbA1c data
- Page 31: Supplementary Clinical Sequelae 1: Hypoglycaemic events
- Page 32: Supplementary Results Description 1

**Supplementary Figure 1:** Change in HbA1c & Body Weight Compared to Randomisation

**Panel 1: HbA1c**


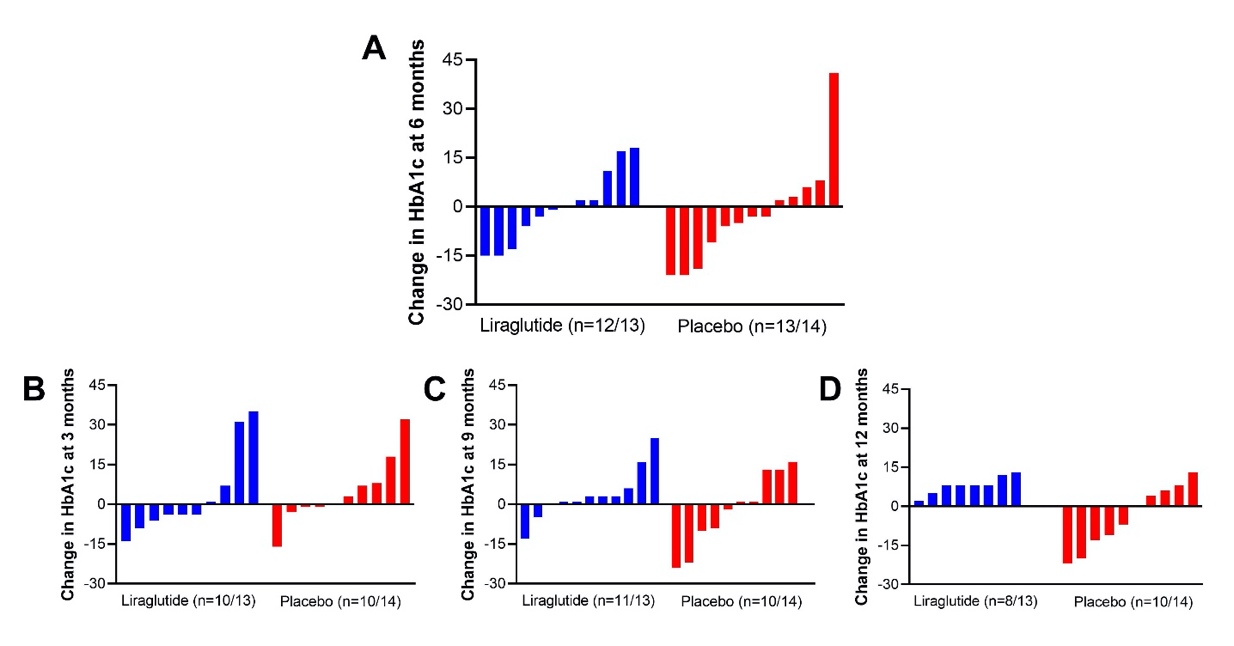


**Panel 2: Body Weight**


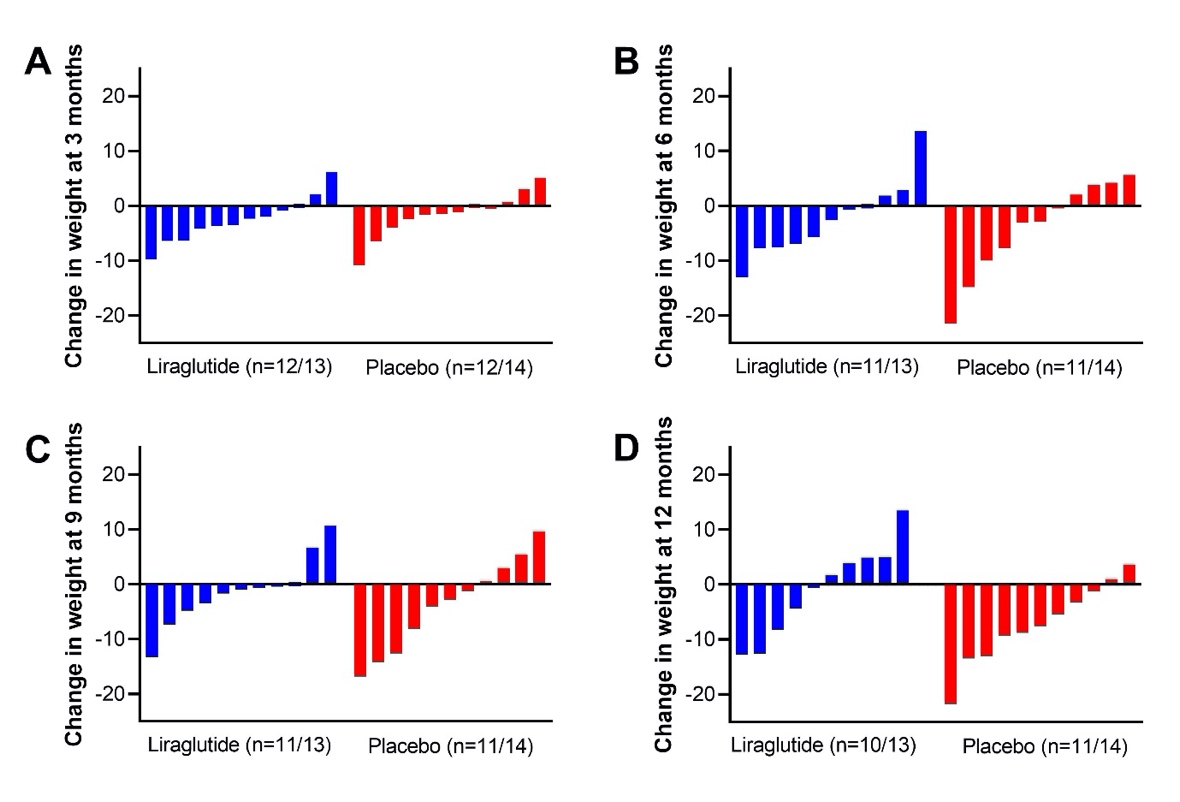


**Panel 1 – HbA1c:** Graphs showing Individual Changes in HbA1c compared to randomisation

A – 6 months, B- 3 months, C - 9 months, D – 12 months

**Panel 2 – Body Weight:** Graphs showing Individual Change in Body Weight compared to randomisation

A – 3 months, B – 6 months, C – 9 months, D – 12 months

**Supplementary Figure 2: Descriptive analysis of length of T2DM**


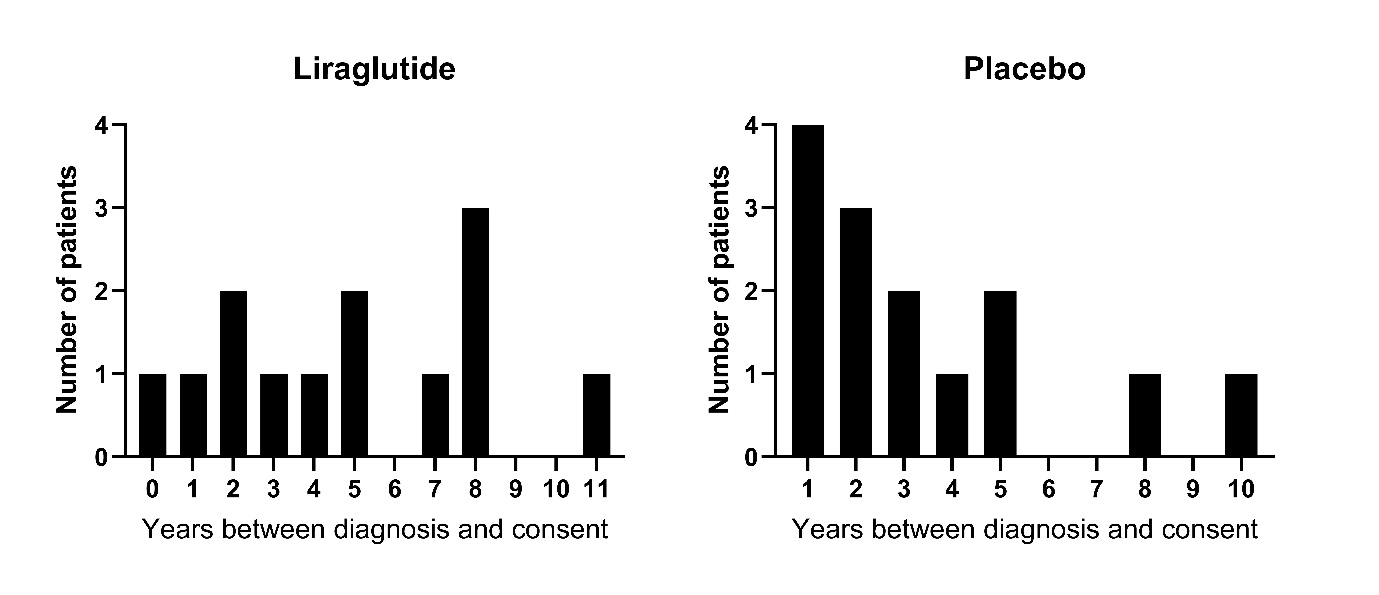


Figure 1: Years between T2DM diagnosis and providing consent for GLIDE in each treatment arm.

Overall patients had a T2DM diagnosis for a median of 3 years (interquartile range (IQR): 2-7 years). This ranged from 0 years to 11 years. Within the Liraglutide arm only, patients had a median duration of T2DM for 5 years (IQR: 2-8 years) ranging from 0 years to 11 years. There was a smaller variation in the Placebo arm with a median duration of 2.5 years (IQR: 1-5 years) and a full range of 1 years to 10 years. The distribution of T2DM durations seemed to differ between treatment arms with a positive skew in the Placebo arm and a more uniform distribution in the Liraglutide arm (Figure 1). Comparisons of these distributions showed no significant difference (p=0.23, examined using a Mann-Whitney test).

**Supplementary Figure 3: Patient Trajectories for HbA1c and Body Weight**

**Supplementary Figure 3.1: Patient Trajectories for HbA1c in each treatment group**


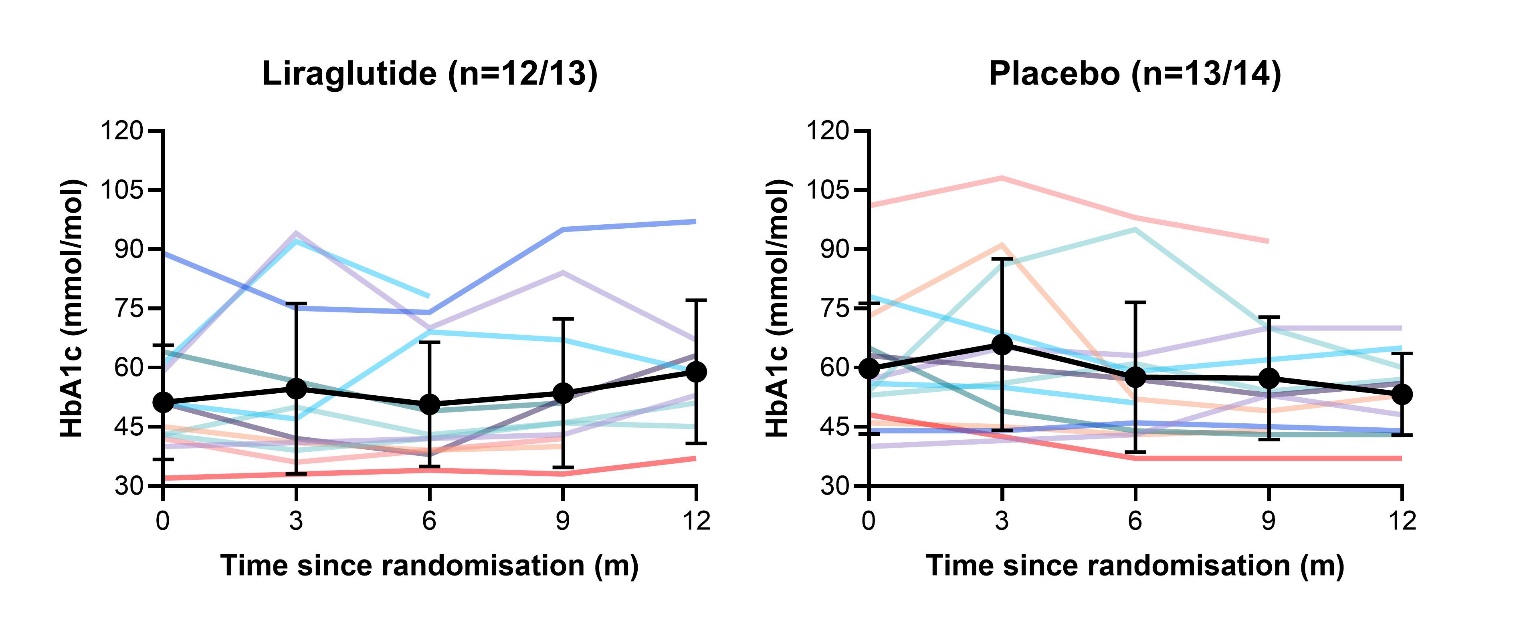


Treatment group average is the black overlay. Two patients (one in each group) did not have any recorded HbA1c measures after baseline and so have been excluded from this graph. M = month

**Supplementary Figure 3.2: Patient Trajectories for Body Weight within each treatment group**


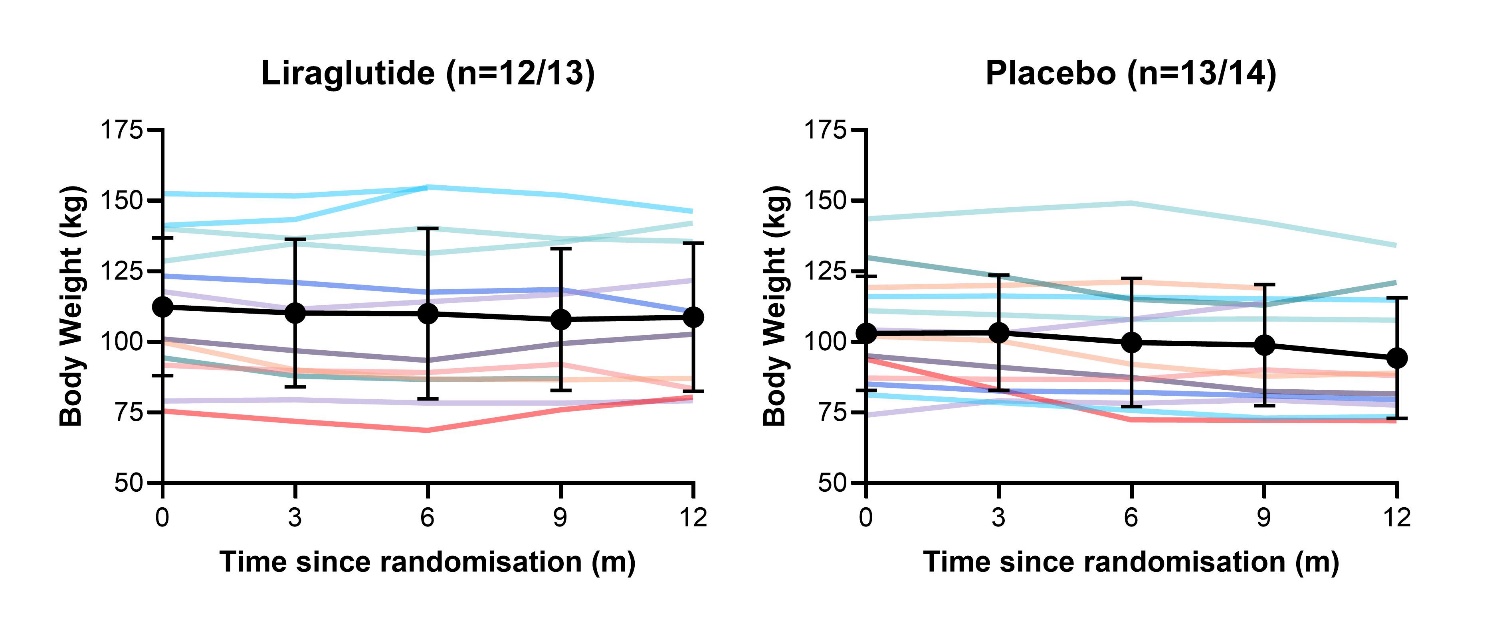


treatment group average is the black overlay. Two patients (one in each group) did not have any recorded weight measures after baseline and so have been excluded from this graph.

**Supplementary Figure 4: Scatter Plot of Relationship between Change in HbA1c (y axis) and change in weight (x-axis)**


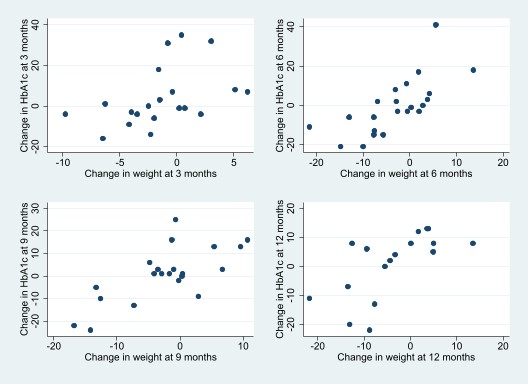


3 months (top left), 6 months (top right), 9 months (bottom left) and 12 months (bottom right)

**Table to Aid interpretation of Supplementary Figure 4: Correlations between change in HbA1c and change in weight at each timepoint**

| Timepoint | Num. of obs. | Correlation coefficient | p-value |
| --- | --- | --- | --- |
| 3 months | 20 | 0.598 | ***0.005*** |
| 6 months | 22 | 0.783 | ***<0.001*** |
| 9 months | 21 | 0.601 | ***0.004*** |
| 12 months | 17 | 0.670 | ***0.003*** |

**Supplementary Figure 5:** Boxplots of HbA1c & Body Weight Change over 12 months


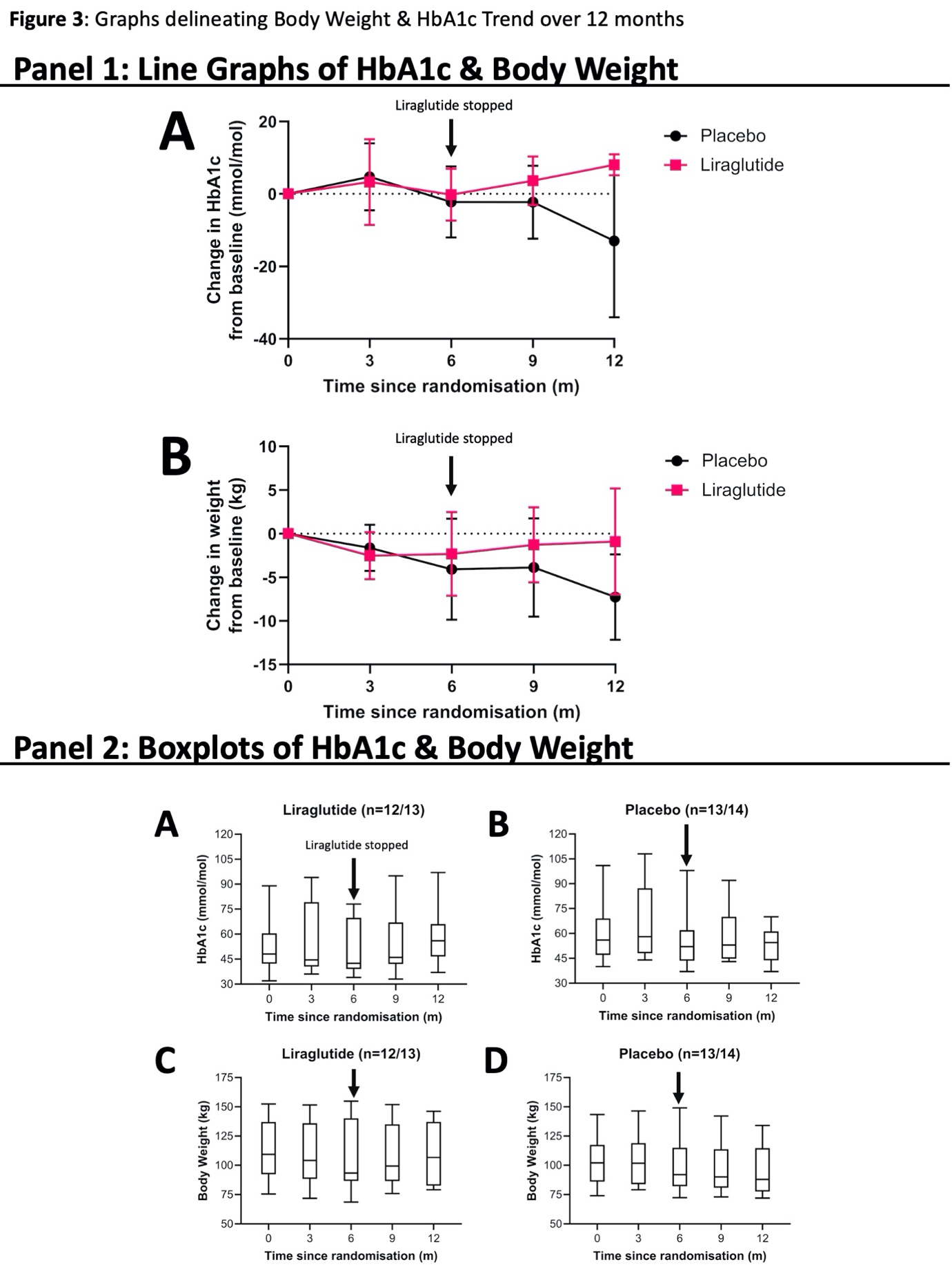


Data is presented as median and interquartile range. Arrow represents when liraglutide / placebo was stopped.

A: Liraglutide HbA1c boxplots, B: Placebo HbA1c boxplots, C: Liraglutide Body Weight boxplots, D: placebo body weight boxplots. M = month

**Supplementary Table 1: Inclusion and Exclusion Criteria**

| Number | Inclusion Criteria | Exclusion Criteria |
| --- | --- | --- |
| 1 | Adult patients with type 2 diabetes diagnosed within the last 10 years | Patients with type 1 diabetes (or thought likely to have type 1 diabetes based on clinical judgement) |
| 2 | Age 18-70 years | Patients who refuse or are unable to have injectable treatment post operatively |
| 3 | HbA1c ≥6.5% and <11% at or before screening | Patients with any disability preventing use of treatment |
| 4 | Patients with a BMI equal to or above 30 kg/m2 (or 27.5kg/m2 and of Asian family origin) and less than or equal to 50kg/m2 at time of screening | Patients with known delayed gastric emptying (diagnosed by clinical history and judgment) |
| 5 | Patients undergoing LAGB based on NICE criteria and multidisciplinary assessment | Patients with any hypersensitivity to liraglutide or any of the excipients listed in the summary of product characteristics; or any limitation to liraglutide use (active at time of screening or likely to be recurrent and/or clinically significant in the future) as per the summary of product characteristics (for example: Inflammatory bowel disease, Diabetic Ketoacidosis, diabetic gastroparesis (based on clinical assessment), severe renal impairment (eGFR < 30mL/min/1.73m2), severe hepatic impairment, acute pancreatitis (persistent, severe abdominal pain) and congestive heart failure NYHA class IV) |
| 6 | Written informed consent to participate. | Type 2 diabetes controlled purely through diet unless metformin is contraindicated or not tolerated |
| 7 |  | Pregnancy or breastfeeding or planning to become pregnant during the study period and women of childbearing age who are not using adequate contraceptive methods (defined as: established use of oral, injected or implanted hormonal methods of contraception; placement of intrauterine device or intrauterine system; barrier methods of contraception (condom or occlusive cap with spermicidal foam/gel/film/cream/suppository); female sterilisation; male sterilisation (where partner is the sole partner of subject); true abstinence (when in line with preferred and usual lifestyle) |
| 8 |  | Personal or family history of thyroid cancer or multiple endocrine neoplasia |
| 9 |  | History of previous pancreatitis |
| 10 |  | Administration of a GLP-1 agonist or DPP-IV inhibitor after surgery |
| 11 |  | Patients who display insufficient understanding of the trial procedures following reasonable attempts by the investigator to provide information, at the discretion of the investigator |

**Supplementary Table 2: Medical History**

|  | Total (n=27) | Liraglutide (n=13) | Placebo (n=14) | p-value |
| --- | --- | --- | --- | --- |
| Hypertension | 18 (66.7%) | 8 (61.5%) | 10 (71.4%) | 0.69 |
| Dyslipidaemia | 16 (61.5%) | 7 (58.3%) | 9 (64.3%) | 1.00 |
| *Missing* | *1* | *1* | *0* |  |
| OSA | 6 (22.2%) | 4 (30.8%) | 2 (14.3%) | 0.38 |
| Arthritis | 7 (25.9%) | 4 (30.8%) | 3 (21.4%) | 0.68 |
| Hormonal contraceptive | 5 (23.8%) | 1 (10.0%) | 4 (36.4%) | 0.31 |
| Diabetes nephropathy | 2 ( 7.4%) | 0 ( 0.0%) | 2 (14.3%) | 0.48 |
| Diabetes neuropathy | 2 ( 7.7%) | 0 ( 0.0%) | 2 (14.3%) | 0.48 |
| Diabetic retinopathy | 2 ( 7.4%) | 0 ( 0.0%) | 2 (14.3%) | 0.48 |
| Erectile dysfunction | 0 ( 0.0%) | 0 ( 0.0%) | 0 ( 0.0%) | - |
| Ischaemic heart disease | 1 ( 3.7%) | 0 ( 0.0%) | 1 ( 7.1%) | 1.00 |
| Myocardial infarction | 0 ( 0.0%) | 0 ( 0.0%) | 0 ( 0.0%) | - |
| Coronary angioplasty | 0 ( 0.0%) | 0 ( 0.0%) | 0 ( 0.0%) | - |
| CABG | 0 ( 0.0%) | 0 ( 0.0%) | 0 ( 0.0%) | - |
| Cardiac failure | 0 ( 0.0%) | 0 ( 0.0%) | 0 ( 0.0%) | - |
| Stroke/TIA | 0 ( 0.0%) | 0 ( 0.0%) | 0 ( 0.0%) | - |
| COPD | 0 ( 0.0%) | 0 ( 0.0%) | 0 ( 0.0%) | - |
| Polycystic ovarian syndrome | 3 (15.0%) | 0 ( 0.0%) | 3 (27.3%) | 0.22 |
| *Missing* | *1* | *1* | *0* |  |

Variables with missing data are indicated. All variables are reported as n(%) and differences between treatment groups were examined using Fisher’s exact test. COPD = chronic obstructive pulmonary disease, OSA = obstructive sleep apnoea, n = number, TIA = transient ischaemic attack.

**Supplementary Table 3: Concomitant medication at date of randomisation and additional medication during follow-up**

|  | Total (n=27) | Liraglutide (n=13) | Placebo (n=14) | p-value |
| --- | --- | --- | --- | --- |
| *At Randomisation* |  |  |  |  |
| Average number of con meds  (per patient) | 6.00 (5.00-9.00) | 6.00 (5.00-7.00) | 7.50 (6.00-9.00) | 0.31 |
| Total recorded number of con meds (across patients) | 193 | 87 | 106 | n/a |
| Number of con meds by system organ class | |  |  | n/a |
| Cardiovascular | 47 | 20 | 27 |  |
| Respiratory | 11 | 8 | 3 |  |
| Gastro-intestinal | 19 | 9 | 10 |  |
| Genito-urinary/renal | 2 | 0 | 2 |  |
| Endocrine | 40 | 16 | 24 |  |
| Musculo-skeletal | 13 | 9 | 4 |  |
| Neurological | 2 | 0 | 2 |  |
| Psychological | 15 | 7 | 8 |  |
| Dermatological | 3 | 3 | 0 |  |
| Allergies | 4 | 0 | 4 |  |
| Food supplement | 25 | 6 | 19 |  |
| Other | 12 | 9 | 3 |  |
| *During study follow-up* |  |  |  |  |
| Average number of new con meds  (per patient) | 1.00 (0.00-3.00) | 2.00 (1.00-3.00) | 0.50 (0.00-2.00) | 0.35 |
| Total recorded number of new con meds (across patients) | 43 | 22 | 21 | n/a |
| Number of new con meds by system organ class | |  |  | n/a |
| Cardiovascular | 4 | 3 | 1 |  |
| Respiratory | 1 | 1 | 0 |  |
| Gastro-intestinal | 8 | 0 | 8 |  |
| Genito-urinary/renal | 2 | 1 | 1 |  |
| Endocrine | 9 | 6 | 3 |  |
| Haematological | 1 | 1 | 0 |  |
| Musculo-skeletal | 6 | 4 | 2 |  |
| Psychological | 2 | 2 | 0 |  |
| Immunological | 2 | 1 | 1 |  |
| Food supplement | 5 | 0 | 5 |  |
| Other | 3 | 3 | 0 |  |

Average number of concomitant medication (con meds) at randomisation and new during follow-up is reported as median (interquartile range) and differences between treatment groups were examined using Wilcoxon rank-sum test. Total number of con meds and system organ class are reported as number of recorded entries. n/a=Differences between treatment groups were not examined. N = number.

**Supplementary Table 4: Concomitant glucose control medication at date of randomisation and additional medication during follow-up**

|  | Total (n=27) | Liraglutide (n=13) | Placebo (n=14) | p-value |
| --- | --- | --- | --- | --- |
| *At Randomisation* |  |  |  |  |
| Average number of glucose meds  (per patient) | 1.00 (1.00-1.00) | 1.00 (1.00-1.00) | 1.00 (1.00-2.00) | 0.081 |
| Patients on Metformin | 26 (96.3%) | 13 (100.0%) | 13 (92.9%) | 1.00 |
| Total recorded number of glucose meds (across patients) | 34 | 14 | 20 | n/a |
| Other Glucose Medications |  |  |  | n/a |
| Gliclazide | 1 | 0 | 1 |  |
| Humulin | 1 | 0 | 1 |  |
| Tresiba Dagludec | 1 | 1 | 0 |  |
| Insulutard | 1 | 0 | 1 |  |
| Glucophage | 1 | 0 | 1 |  |
| Dapagliflozin | 2 | 0 | 2 |  |
| Victoza | 1 | 0 | 1 |  |
| *During study follow-up* |  |  |  |  |
| Average number of new glucose meds  (per patient) | 0.00 (0.00-0.00) | 0.00 (0.00-1.00) | 0.00 (0.00-0.00) | 0.32 |
| Total recorded number of new glucose meds (across patients) | 9 | 6 | 3 | n/a |
| New Glucose Medications |  |  |  | n/a |
| Metformin | 3 | 2 | 1 |  |
| Empagliflozin | 2 | 2 | 0 |  |
| Dapagliflozin | 2 | 0 | 2 |  |
| Canagliflozin | 1 | 1 | 0 |  |
| Semaglutide | 1 | 1 | 0 |  |

Average number of glucose medications (glucose meds) at randomisation and new during follow-up is reported as median (interquartile range) and differences between treatment groups were examined using Wilcoxon rank-sum test. The number of patients on Metformin at randomisation is reported as n(%) and examined using a Fisher’s exact test. Total number of glucose meds and each drug are reported as number of recorded entries. n/a=Differences between treatment groups were not examined. N = number.

**Supplementary Table 5: Multivariate Analysis of HbA1c Across All Time Points**

*Table 5.1: Multivariate analysis of difference in HbA1c at 3 months*

| Variable | Effect estimate | F statistic | p-value |
| --- | --- | --- | --- |
| Liraglutide (vs. Placebo) | -1.69 (-17.16, 13.77) | 0.06 | 0.82 |
| Baseline HbA1c | 1.07 (0.50, 1.64) | 16.91 | ***0.001*** |
| BMI>42 (vs. BMI≤42) | -5.35 (-24.10, 13.39) | 0.39 | 0.55 |
| Duration >5 years (vs. ≤5 years) | 10.73 (-8.21, 29.67) | 1.52 | 0.24 |
| Insulin use (vs. no use) | -9.30 (-34.76, 16.15) | 0.63 | 0.44 |
| Site (vs. GSTT) |  | 1.59 | 0.24 |
| BHH | 12.05 (-11.42, 35.52) |  |  |
| North Bristol | 15.75 (-4.65, 36.16) |  |  |

Table 5.2: Multivariate analysis of difference in HbA1c at 6 months

| Variable | Effect estimate | F statistic | p-value |
| --- | --- | --- | --- |
| Liraglutide (vs. Placebo) | 0.15 (-11.28-11.59) | 0.00 | 0.98 |
| 0-month HbA1c | 0.83 (0.45-1.21) | 21.39 | ***<0.001*** |
| BMI>42 (vs. BMI≤42) | 0.33(-14.87-15.52) | 0.00 | 0.96 |
| Duration >5 years (vs. ≤5 years) | -3.26 (-15.68-9.17) | 0.31 | 0.59 |
| Insulin use (vs. no use) | 3.35 (-17.28-23.99) | 0.12 | 0.74 |
| Site (vs. GSTT) |  | 2.60 | 0.10 |
| BHH | 8.20 (-8.02-24.41) |  |  |
| North Bristol | 17.11 (0.75-33.47) |  |  |

*Table 5.3: Multivariate analysis of difference in HbA1c at 9 months*

| Variable | Effect estimate | F statistic | p-value |
| --- | --- | --- | --- |
| Liraglutide (vs. Placebo) | 5.67 (-4.14, 15.47) | 1.56 | 0.23 |
| Baseline HbA1c | 0.85 (0.53, 1.17) | 33.41 | ***<0.001*** |
| BMI>42 (vs. BMI≤42) | -2.09 (-16.11, 11.93) | 0.10 | 0.75 |
| Duration >5 years (vs. ≤5 years) | -4.09 (-15.86, 7.68) | 0.56 | 0.47 |
| Insulin use (vs. no use) | 11.62 (-4.54, 27.78) | 2.41 | 0.14 |
| Site (vs. GSTT) |  | 7.07 | ***0.008*** |
| BHH | 25.84 (9.91, 41.76) |  |  |
| North Bristol | 10.89 (-2.73, 24.51) |  |  |

Table 5.4: Multivariate analysis of difference in HbA1c at 12 months

| Variable | Effect estimate | F statistic | p-value |
| --- | --- | --- | --- |
| Liraglutide (vs. Placebo) | 10.85 (1.14-20.56) | 6.20 | ***0.032*** |
| 0-month HbA1c | 0.81 (0.38-1.25) | 17.29 | ***0.002*** |
| BMI>42 (vs. BMI≤42) | -6.59 (-20.83-7.64) | 1.07 | 0.33 |
| Duration >5 years (vs. ≤5 years) | -8.74 (-21.49-4.01) | 2.33 | 0.16 |
| Insulin use (vs. no use) | 7.39 (-9.53-24.31) | 0.95 | 0.35 |
| Site (vs. GSTT) |  | 1.12 | 0.36 |
| BHH | 8.98 (-5.08-23.04) |  |  |
| North Bristol | 4.16 (-9.38-17.71) |  |  |

F statistics/ p-values come from ANCOVA including each variable included in the table. Effect estimates comes from multivariate linear regression models with the same covariates. BMI = Body Mass Index, GSTT = Guys and St Thomas’, BHH = Birmingham Heartlands Hospital

**Supplementary Table 6: Multivariate Analysis of Body Weight Change at All Time Points**

Table 6.1: Multivariate analysis of difference in body weight at 3 months

| Variable | Effect estimate | F statistic | p-value |
| --- | --- | --- | --- |
| Liraglutide (vs. Placebo) | -0.05 (-3.02, 2.91) | 0.00 | 0.97 |
| Baseline body weight | 0.93 (0.81, 1.05) | 269.05 | ***<0.001*** |
| BMI>42 (vs. BMI≤42) | 2.97 (-2.05, 7.99) | 1.57 | 0.23 |
| Duration >5 years (vs. ≤5 years) | -1.39 (-4.48, 1.71) | 0.90 | 0.36 |
| Insulin use (vs. no use) | 3.06 (-2.20, 8.32) | 1.52 | 0.24 |
| Site (vs. GSTT) |  | 5.97 | ***0.012*** |
| BHH | 5.52 (0.60, 10.45) |  |  |
| North Bristol | 5.91 (1.14, 10.68) |  |  |

Table 6.2: Multivariate analysis of difference in body weight at 6 months

| Variable | Effect estimate | F statistic | p-value |
| --- | --- | --- | --- |
| Liraglutide (vs. Placebo) | 1.98 (-4.18-8.14) | 0.47 | 0.50 |
| Baseline body weight | 0.95 (0.69-1.21) | 59.29 | ***<0.001*** |
| BMI>42 (vs. BMI≤42) | 2.05 (-8.58-12.67) | 0.17 | 0.69 |
| Duration >5 years (vs. ≤5 years) | -4.40 (-10.97-2.17) | 2.06 | 0.17 |
| Insulin use (vs. no use) | 4.34 (-6.38-15.07) | 0.76 | 0.40 |
| Site (vs. GSTT) |  | 4.03 | ***0.041*** |
| BHH | 9.10 (-2.61-20.82) |  |  |
| North Bristol | 10.15 (-0.62-20.92) |  |  |

Table 6.3: Multivariate analysis of difference in body weight at 9 months

| Variable | Effect estimate | F statistic | p-value |
| --- | --- | --- | --- |
| Liraglutide (vs. Placebo) | 4.58 (-2.25, 11.41) | 2.07 | 0.17 |
| Baseline body weight | 0.83 (0.55, 1.11) | 39.90 | ***<0.001*** |
| BMI>42 (vs. BMI≤42) | 2.03 (-9.55, 13.62) | 0.14 | 0.71 |
| Duration >5 years (vs. ≤5 years) | -6.72 (-14.70, 1.27) | 3.25 | 0.093 |
| Insulin use (vs. no use) | 2.48 (-9.37, 14.33) | 0.20 | 0.66 |
| Site (vs. GSTT) |  | 1.59 | 0.24 |
| BHH | 3.18 (-8.35, 14.72) |  |  |
| North Bristol | 8.31 (-2.99, 19.62) |  |  |

Table 6.4: Multivariate analysis of difference in body weight at 12 months

| Variable | Effect estimate | F statistic | p-value |
| --- | --- | --- | --- |
| Liraglutide (vs. Placebo) | 8.23 (1.55-14.92) | 7.09 | ***0.020*** |
| Baseline body weight | 0.75 (0.47-1.03) | 33.47 | ***<0.001*** |
| BMI>42 (vs. BMI≤42) | 9.98 (-1.43-21.39) | 3.57 | 0.081 |
| Duration >5 years (vs. ≤5 years) | -5.82 (-13.36-1.71) | 2.79 | 0.12 |
| Insulin use (vs. no use) | 0.43 (-10.89-11.76) | 0.01 | 0.94 |
| Site (vs. GSTT) |  | 1.72 | 0.22 |
| BHH | 4.98 (-5.02-14.98) |  |  |
| North Bristol | 7.30 (-3.67-18.27) |  |  |

F statistics/ p-values come from ANCOVA including each variable included in the table. Effect estimates comes from multivariate linear regression models with the same covariates. BMI = Body Mass Index, GSTT = Guys and St Thomas’, BHH = Birmingham Heartlands Hospital

**Supplementary Table 7: Multivariate Analysis of Percentage Body Weight Change at All Timepoints**

Table 7.1: Multivariate analysis of percentage change in body weight at 3 months

| Variable | Effect estimate | F statistic | p-value |
| --- | --- | --- | --- |
| Liraglutide (vs. Placebo) | -0.57 (-3.33, 2.20) | 0.19 | 0.67 |
| BMI>42 (vs. BMI≤42) | 0.86 (-2.98, 4.69) | 0.22 | 0.64 |
| Duration >5 years (vs. ≤5 years) | -2.01 (-5.00, 0.97) | 2.02 | 0.17 |
| Insulin use (vs. no use) | 2.59 (-2.42, 7.61) | 1.19 | 0.29 |
| Site (vs. GSTT) |  | 7.30 | ***0.005*** |
| BHH | 7.48 (3.07, 11.88) |  |  |
| North Bristol | 4.37 (0.27, 8.46) |  |  |

Table 7.2: Multivariate analysis of percentage change in body weight at 6 months

| Variable | Effect estimate | F statistic | p-value |
| --- | --- | --- | --- |
| Liraglutide (vs. Placebo) | 1.24 (-4.05, 6.52) | 0.25 | 0.63 |
| BMI>42 (vs. BMI≤42) | 1.48 (-6.38, 9.33) | 0.16 | 0.69 |
| Duration >5 years (vs. ≤5 years) | -5.11 (-10.85, 0.64) | 3.59 | 0.078 |
| Insulin use (vs. no use) | 4.46 (-4.76, 13.68) | 1.06 | 0.32 |
| Site (vs. GSTT) |  | 4.80 | ***0.025*** |
| BHH | 10.87 (1.65, 20.10) |  |  |
| North Bristol | 7.81 (-0.03, 15.64) |  |  |

Table 7.3: Multivariate analysis of percentage change in body weight at 9 months

| Variable | Effect estimate | F statistic | p-value |
| --- | --- | --- | --- |
| Liraglutide (vs. Placebo) | 3.09 (-3.20, 9.38) | 1.10 | 0.31 |
| BMI>42 (vs. BMI≤42) | -0.87 (-10.60, 8.85) | 0.04 | 0.85 |
| Duration >5 years (vs. ≤5 years) | -7.14 (-14.68, 0.41) | 4.06 | 0.062 |
| Insulin use (vs. no use) | 1.25 (-9.83, 12.33) | 0.06 | 0.81 |
| Site (vs. GSTT) |  | 1.15 | 0.34 |
| BHH | 6.08 (-3.68, 15.85) |  |  |
| North Bristol | 3.73 (-5.64, 13.09) |  |  |

Table 7.4: Multivariate analysis of percentage change in body weight at 12 months

| Variable | Effect estimate | F statistic | p-value |
| --- | --- | --- | --- |
| Liraglutide (vs. Placebo) | 6.63 (-0.35, 13.60) | 4.16 | 0.061 |
| BMI>42 (vs. BMI≤42) | 3.33 (-5.85, 12.52) | 0.61 | 0.45 |
| Duration >5 years (vs. ≤5 years) | -6.36 (-14.40, 1.68) | 2.88 | 0.11 |
| Insulin use (vs. no use) | -0.73 (-12.65, 11.19) | 0.02 | 0.90 |
| Site (vs. GSTT) |  | 1.82 | 0.20 |
| BHH | 8.62 (-1.07, 18.32) |  |  |
| North Bristol | 2.05 (-8.02, 12.11) |  |  |

F statistics/ p-values come from ANCOVA including each variable included in the table. Effect estimates comes from multivariate linear regression models with the same covariates. BMI = Body Mass Index, GSTT = Guys and St Thomas’, BHH = Birmingham Heartlands Hospital

**Supplementary Table 8: Remission of diabetes**

|  | Total (n=27) | Liraglutide (n=13) | Placebo (n=14) | p-value |
| --- | --- | --- | --- | --- |
| Diabetes in remission at… | |  |  |  |
| 3 months | 1 ( 4.2%) | 1 ( 8.3%) | 0 ( 0.0%) | 1.00 |
| *Missing* | *3* | *1* | *2* |  |
| 6 months | 3 (13.6%) | 1 ( 9.1%) | 2 (18.2%) | 1.00 |
| *Missing* | *5* | *2* | *3* |  |
| 9 months | 4 (18.2%) | 2 (18.2%) | 2 (18.2%) | 1.00 |
| *Missing* | *5* | *2* | *3* |  |
| 12 months | 6 (26.1%) | 3 (27.3%) | 3 (25.0%) | 1.00 |
| *Missing* | *4* | *2* | *2* |  |

Missing data is indicated. Data for all timepoints is reported as n(%). Differences between treatment groups were examined using Fisher’s exact test. N = number.

**Supplementary Table 9: Measures of Diabetes**

|  | Total (n=27) | Liraglutide (n=13) | Placebo (n=14) | p-value |
| --- | --- | --- | --- | --- |
| Fasting glucose (mmol/L) |  |  |  |  |
| Baseline | 6.9 (6.4-8.8) | 6.7 (5.5-8.5) | 7.2 (6.7-9.8) | 0.14 |
| *Missing* | *3* | *1* | *2* |  |
| 3 months |  |  |  |  |
| Average | 7.7 (6.5-12.2) | 7.0 (5.3-13.3) | 7.7 (7.1-12.2) | 0.53 |
| Change from baseline | 0.3 (-0.3-1.3) | 0.4 (-0.4-3.3) | 0.3 (-0.1-0.9) | 0.67 |
| *Missing* | *9* | *5* | *4* |  |
| 6 months |  |  |  |  |
| Average | 6.6 (5.7-9.4) | 6.1 (5.1-9.4) | 6.7 (6.0-9.6) | 0.41 |
| Change from baseline | -0.6 (-2.1-0.4) | -0.3 (-1.3-0.4) | -1.4 (-2.8--0.3) | 0.15 |
| *Missing* | *5* | *2* | *3* |  |
| 9 months |  |  |  |  |
| Average | 6.5 (5.9-7.6) | 6.6 (5.8-9.9) | 6.2 (5.9-7.5) | 0.34 |
| Change from baseline | -0.2 (-1.2-1.5) | 1.4 (-0.6-1.6) | -1.7 (-6.6--0.2) | 0.011 |
| *Missing* | *7* | *2* | *5* |  |
| 12 months |  |  |  |  |
| Average | 7.2 (6.0-9.4) | 7.5 (5.9-10.1) | 6.8 (6.3-8.4) | 0.60 |
| Change from baseline | -0.7 (-1.9-0.3) | 0.2 (-0.8-1.1) | -1.9 (-3.1--0.7) | 0.037 |
| *Missing* | *12* | *6* | *6* |  |
| Fasting insulin (pmol/L) |  |  |  |  |
| Baseline | 120.0 (86.0-150.0) | 120.0 (86.0-157.0) | 116.0 (84.0-138.0) | 0.53 |
| *Missing* | *6* | *2* | *4* |  |
| 3 months |  |  |  |  |
| Average | 127.0 (90.0-179.0) | 127.0 (117.0-399.0) | 130.5 (85.0-164.0) | 0.49 |
| Change from baseline | 15.0 (-2.5-35.5) | 35.5 (24.0-284.0) | 2.0 (-4.0-6.0) | 0.045 |
| *Missing* | *10* | *6* | *4* |  |
| 6 months |  |  |  |  |
| Average | 105.0 (81.0-158.0) | 92.0 (81.0-311.0) | 108.0 (61.0-137.0) | 0.60 |
| Change from baseline | -8.0 (-31.0-42.0) | 17.0 (-23.0-88.0) | -17.5 (-31.0--8.0) | 0.22 |
| *Missing* | *6* | *2* | *4* |  |
| 9 months |  |  |  |  |
| Average | 117.0 (84.0-200.0) | 126.0 (81.0-224.0) | 117.0 (103.0-168.0) | 0.71 |
| Change from baseline | 6.0 (-1.0-55.0) | 7.5 (2.5-58.5) | 3.0 (-52.0-55.0) | 0.46 |
| *Missing* | *8* | *3* | *5* |  |
| 12 months |  |  |  |  |
| Average | 116.0 (76.0-134.0) | 106.5 (76.0-194.0) | 116.0 (38.0-134.0) | 0.57 |
| Change from baseline | -26.0 (-44.0-29.0) | 21.5 (-26.0-37.0) | -46.0 (-84.0--43.0) | 0.039 |
| *Missing* | *14* | *7* | *7* |  |
| HOMA-IR |  |  |  |  |
| Baseline | 6.5 (5.3-8.4) | 6.2 (5.5-9.6) | 6.8 (5.1-8.2) | 0.71 |
| *Missing* | *7* | *3* | *4* |  |
| 3 months |  |  |  |  |
| Average | 7.8 (4.9-10.4) | 5.0 (4.8-34.6) | 8.3 (7.2-10.4) | 0.56 |
| Change from baseline | 0.7 (0.3-1.3) | 1.3 (0.3-25.0) | 0.4 (0.3-1.0) | 0.11 |
| *Missing* | *10* | *6* | *4* |  |
| 6 months |  |  |  |  |
| Average | 4.8 (3.4-11.2) | 4.7 (3.1-24.2) | 5.0 (4.0-10.2) | 0.89 |
| Change from baseline | -0.6 (-3.5-3.6) | 0.6 (-2.5-11.2) | -2.1 (-4.0--0.5) | 0.093 |
| *Missing* | *6* | *2* | *4* |  |
| 9 months |  |  |  |  |
| Average | 5.9 (4.0-10.8) | 5.7 (4.5-16.4) | 5.9 (3.7-7.3) | 0.46 |
| Change from baseline | 0.1 (-1.2-3.9) | 1.1 (-0.4-8.1) | -1.1 (-4.5--0.0) | 0.088 |
| *Missing* | *8* | *3* | *5* |  |
| 12 months |  |  |  |  |
| Average | 6.2 (3.3-6.9) | 6.9 (3.3-11.8) | 5.4 (1.8-6.5) | 0.25 |
| Change from baseline | -2.7 (-4.0-3.4) | 3.4 (-2.6-3.4) | -5.1 (-5.3--2.8) | 0.053 |
| *Missing* | *14* | *7* | *7* |  |

Missing data is indicated. Data for all timepoints is reported as Median (Interquartile Range) due to a number of non-normally distributed variables. Differences between treatment groups were examined using Wilcoxon rank-sum test. HOMA-IR = Homeostatic Model Assessment for Insulin Resistance, n = number.

**Supplementary Table 10: Multivariate Analysis of Fasting Glucose**

**Supplementary Table 10.1 : Multivariate analysis of fasting glucose at 6 months**

| Variable | Effect estimate | F statistic | p-value |
| --- | --- | --- | --- |
| Liraglutide (vs. Placebo) | 1.43 (-1.09, 3.94) | 1.56 | 0.24 |
| Baseline fasting glucose | 0.69 (0.07, 1.31) | 6.01 | ***0.032*** |
| BMI>42 (vs. BMI≤42) | 2.16 (-1.69, 6.01) | 1.52 | 0.24 |
| Duration >5 years (vs. ≤5 years) | -0.58 (-3.40, 2.24) | 0.20 | 0.66 |
| Insulin use (vs. no use) | 0.92 (-2.93, 4.77) | 0.28 | 0.61 |
| Site (vs. GSTT) |  | 0.25 | 0.78 |
| BHH | 0.53 (-2.89, 3.95) |  |  |
| North Bristol | -1.11 (-5.14, 2.93) |  |  |

The F statistic and p-value come from an ANCOVA model including each model included in the table. Estimated effects of each variable on fasting glucose at 6 months comes from a multivariate linear regression with the same covariates. BMI = Body Mass Index.

**Supplementary Table 10.2: Multivariate analysis of fasting glucose at 12 months**

| Variable | Effect estimate | F statistic | p-value |
| --- | --- | --- | --- |
| Liraglutide (vs. Placebo) | 2.24 (0.43, 4.06) | 11.78 | ***0.027*** |
| Baseline fasting glucose | 0.15 (-0.53, 0.83) | 0.38 | 0.57 |
| BMI>42 (vs. BMI≤42) | 1.65 (-1.09, 4.39) | 2.78 | 0.17 |
| Duration >5 years (vs. ≤5 years) | 1.11 (-1.98, 4.20) | 1.00 | 0.37 |
| Insulin use (vs. no use) | 1.25 (-1.23, 3.72) | 1.95 | 0.24 |
| Site (vs. GSTT) |  | 2.51 | 0.20 |
| BHH | 1.85 (-1.13, 4.83) |  |  |
| North Bristol | -0.98 (-3.63, 1.66) |  |  |

The F statistic and p-value come from an ANCOVA model including each model included in the table. Estimated effects of each variable on fasting glucose at 12 months comes from a multivariate linear regression with the same covariates.

**Supplementary Table 11: Anthropometric measures and Body Composition across follow-up**

|  | Total (n=27) | Liraglutide (n=13) | Placebo (n=14) | p-value |
| --- | --- | --- | --- | --- |
| Body Mass Index |  |  |  |  |
| Baseline | 37.77 (33.20-43.66) | 38.91 (35.18-46.11) | 34.76 (32.11-42.87) | 0.11 |
| 3 months |  |  |  |  |
| Average | 34.89 (32.45-44.71) | 37.32 (34.06-47.01) | 34.24 (31.83-42.23) | 0.15 |
| Change from baseline | -0.63 (-1.62-0.12) | -0.98 (-1.86--0.04) | -0.42 (-1.16-0.17) | 0.39 |
| *Missing* | *3* | *1* | *2* |  |
| 6 months |  |  |  |  |
| Average | 33.97 (31.50-46.18) | 36.48 (33.54-48.70) | 32.20 (30.93-37.98) | 0.082 |
| Change from baseline | -0.98 (-2.99-0.76) | -0.98 (-2.99-0.60) | -0.98 (-3.38-1.16) | 0.92 |
| *Missing* | *5* | *2* | *3* |  |
| 9 months |  |  |  |  |
| Average | 34.68 (31.60-38.79) | 37.40 (33.89-47.23) | 33.46 (29.68-37.32) | 0.061 |
| Change from baseline | -0.54 (-2.92-0.17) | -0.33 (-1.51-0.17) | -0.92 (-4.26-1.08) | 0.45 |
| *Missing* | *5* | *2* | *3* |  |
| 12 months |  |  |  |  |
| Average | 34.24 (31.05-40.20) | 37.53 (34.24-46.92) | 31.05 (29.97-39.97) | 0.024 |
| Change from baseline | -1.52 (-3.12-0.66) | 0.35 (-3.12-1.63) | -2.91 (-4.43--0.49) | 0.14 |
| *Missing* | *6* | *3* | *3* |  |
| Neck circumference (cm) |  |  |  |  |
| Baseline | 39.0 (37.0-44.0) | 39.0 (37.0-44.0) | 39.0 (37.0-42.0) | 0.73 |
| 3 months |  |  |  |  |
| Average | 38.0 (36.0-44.0) | 37.00 (36.0-45.0) | 39.50 (36.0-43.0) | 0.94 |
| Change from baseline | 0.0 (-2.0-1.0) | 0.00 (-2.0-1.0) | 0.00 (-2.0-1.0) | 0.74 |
| *Missing* | *6* | *2* | *4* |  |
| 6 months |  |  |  |  |
| Average | 36.0 (35.0-43.0) | 36.0 (35.0-44.0) | 36.5 (34.5-37.5) | 0.48 |
| Change from baseline | -1.0 (-3.0-0.0) | -1.0 (-3.0-0.0) | -1.0 (-3.0-0.0) | 0.80 |
| *Missing* | *8* | *2* | *6* |  |
| 9 months |  |  |  |  |
| Average | 37.0 (35.0-44.0) | 36.0 (35.0-47.0) | 38.0 (36.0-44.0) | 0.59 |
| Change from baseline | -1.0 (-3.0-1.0) | -1.0 (-3.0-1.0) | -1.0 (-3.0-1.0) | 0.97 |
| *Missing* | *6* | *2* | *4* |  |
| 12 months |  |  |  |  |
| Average | 38.0 (36.0-45.0) | 37.0 (35.0-45.0) | 40.0 (36.0-45.0) | 0.48 |
| Change from baseline | -1.0 (-3.0-0.0) | 0.0 (-1.0-0.0) | -2.0 (-3.0-1.0) | 0.36 |
| *Missing* | *13* | *6* | *7* |  |
| Waist circumference (cm) |  |  |  |  |
| Baseline | 114.0 (107.0-127.0) | 120.0 (108.0-137.0) | 113.0 (106.0-122.0) | 0.33 |
| 3 months |  |  |  |  |
| Average | 113.0 (109.0-132.0) | 119.0 (105.0-139.0) | 112.5 (109.0-120.0) | 0.75 |
| Change from baseline | -2.0 (-6.0-2.0) | -2.0 (-5.0-2.0) | -1.5 (-6.0-3.0) | 0.65 |
| *Missing* | *6* | *2* | *4* |  |
| 6 months |  |  |  |  |
| Average | 111.0 (102.0-132.0) | 116.00 (103.0-136.0) | 107.50 (99.5-119.0) | 0.46 |
| Change from baseline | -4.0 (-11.0--1.0) | -4.00 (-9.0--1.0) | -6.00 (-12.0-1.5) | 0.68 |
| *Missing* | *8* | *2* | *6* |  |
| 9 months |  |  |  |  |
| Average | 114.0 (101.0-120.0) | 114.0 (101.0-133.0) | 113.0 (101.0-120.0) | 0.86 |
| Change from baseline | -4.0 (-9.0-2.0) | -2.0 (-7.0-2.0) | -7.0 (-10.0-3.0) | 0.46 |
| *Missing* | *6* | *2* | *4* |  |
| 12 months |  |  |  |  |
| Average | 114.0 (99.0-125.0) | 117.0 (99.0-127.0) | 111.0 (98.0-123.0) | 0.52 |
| Change from baseline | -2.5 (-10.0-3.0) | 3.0 (-4.0-3.0) | -10.0 (-18.0--2.0) | 0.16 |
| *Missing* | *13* | *6* | *7* |  |
| Fat free mass (kg) |  |  |  |  |
| Baseline | 55.00 (49.50-67.50) | 56.30 (49.70-67.50) | 53.10 (49.30-66.50) | 0.61 |
| 3 months |  |  |  |  |
| Average | 56.90 (49.00-68.00) | 56.90 (49.00-68.00) | 54.60 (49.00-64.20) | 0.81 |
| Change from baseline | -0.50 (-1.40-0.60) | -0.70 (-4.70-1.00) | -0.50 (-1.40-0.60) | 0.89 |
| *Missing* | *6* | *2* | *4* |  |
| 6 months |  |  |  |  |
| Average | 54.10 (47.70-78.80) | 54.10 (45.60-83.70) | 53.70 (48.55-70.40) | 1.00 |
| Change from baseline | -0.90 (-2.80-1.10) | -1.30 (-2.80-4.00) | -0.70 (-3.10-0.95) | 0.77 |
| *Missing* | *8* | *2* | *6* |  |
| 9 months |  |  |  |  |
| Average | 55.40 (48.35-69.45) | 53.50 (47.20-68.30) | 57.30 (48.80-70.60) | 0.68 |
| Change from baseline | -1.00 (-2.35-0.30) | -0.30 (-2.00-1.80) | -1.50 (-2.40-0.20) | 0.29 |
| *Missing* | *7* | *2* | *5* |  |
| 12 months |  |  |  |  |
| Average | 55.50 (46.70-68.00) | 59.60 (47.50-68.00) | 47.90 (46.30-69.50) | 0.65 |
| Change from baseline | -2.65 (-3.50-0.30) | 0.30 (-3.60-2.60) | -3.30 (-3.50--2.30) | 0.14 |
| *Missing* | *13* | *6* | *7* |  |
| Fat mass (kg) |  |  |  |  |
| Baseline | 46.30 (36.00-60.30) | 46.30 (42.80-61.00) | 47.65 (35.00-56.10) | 0.48 |
| 3 months |  |  |  |  |
| Average | 48.30 (39.70-59.90) | 52.00 (40.20-64.30) | 44.15 (34.90-59.10) | 0.23 |
| Change from baseline | -2.30 (-3.70-0.10) | -2.30 (-3.70-1.50) | -2.40 (-4.20-0.10) | 0.67 |
| *Missing* | *6* | *2* | *4* |  |
| 6 months |  |  |  |  |
| Average | 40.70 (33.10-63.40) | 40.70 (32.20-67.40) | 40.85 (34.65-57.80) | 0.74 |
| Change from baseline | -1.90 (-6.80-1.20) | -1.30 (-5.60-2.20) | -4.85 (-8.55-1.20) | 0.51 |
| *Missing* | *8* | *2* | *6* |  |
| 9 months |  |  |  |  |
| Average | 38.90 (35.90-57.50) | 43.00 (35.40-68.00) | 38.30 (35.90-54.10) | 0.44 |
| Change from baseline | -0.60 (-6.30-1.00) | -0.30 (-6.30-1.20) | -1.30 (-9.10-0.20) | 0.57 |
| *Missing* | *6* | *2* | *4* |  |
| 12 months |  |  |  |  |
| Average | 41.90 (32.80-56.10) | 51.30 (32.30-63.00) | 38.20 (32.80-52.40) | 0.28 |
| Change from baseline | -3.65 (-9.90-1.30) | 1.30 (-4.10-3.30) | -7.20 (-10.10--3.20) | 0.035 |
| *Missing* | *13* | *6* | *7* |  |
| Fat percentage (%) |  |  |  |  |
| Baseline | 45.40 (40.50-48.00) | 45.40 (43.20-47.50) | 44.30 (40.50-48.00) | 0.63 |
| 3 months |  |  |  |  |
| Average | 44.80 (42.30-48.00) | 44.80 (42.50-48.50) | 42.80 (40.90-47.90) | 0.29 |
| Change from baseline | -1.00 (-2.30-0.40) | -1.00 (-2.40-2.00) | -1.10 (-2.30--0.20) | 0.53 |
| *Missing* | *6* | *2* | *4* |  |
| 6 months |  |  |  |  |
| Average | 44.00 (38.30-47.40) | 45.10 (37.30-47.40) | 43.10 (41.25-46.80) | 0.80 |
| Change from baseline | 0.10 (-3.60-1.50) | 0.20 (-3.90-1.50) | -2.25 (-3.20-0.80) | 0.54 |
| *Missing* | *8* | *2* | *6* |  |
| 9 months |  |  |  |  |
| Average | 43.80 (39.80-47.00) | 45.80 (38.30-49.40) | 41.95 (39.80-44.30) | 0.40 |
| Change from baseline | -0.70 (-2.00-1.10) | -0.80 (-2.00-1.20) | -0.55 (-3.20-0.50) | 0.89 |
| *Missing* | *6* | *2* | *4* |  |
| 12 months |  |  |  |  |
| Average | 42.20 (39.10-48.40) | 43.10 (39.20-49.90) | 41.30 (35.50-45.80) | 0.25 |
| Change from baseline | -1.00 (-3.80-1.20) | -0.60 (-1.60-1.90) | -1.70 (-4.50--0.40) | 0.14 |
| *Missing* | *13* | *6* | *7* |  |

For Table above: Missing data is indicated. Data for all timepoints is reported as Median (Interquartile Range) due to a number of non-normally distributed variables. Differences between treatment groups were examined using Wilcoxon rank-sum test. N = number.

**Supplementary Table 12: Cardiovascular Disease Risk Factors across follow-up**

|  | Total (n=27) | Liraglutide (n=13) | Placebo (n=14) | p-value |
| --- | --- | --- | --- | --- |
| Systolic Blood Pressure across 3 sittings (mmHg) | |  |  |  |
| Baseline | 124.00 (114.00-127.00) | 121.00 (111.33-124.67) | 125.67 (123.67-130.00) | 0.012 |
| 3 months |  |  |  |  |
| Average | 116.67 (111.00-126.67) | 115.67 (110.00-125.33) | 124.83 (112.33-136.67) | 0.19 |
| Change from baseline | -3.33 (-6.67-0.67) | -0.67 (-8.67-3.33) | -3.67 (-6.67--0.33) | 0.57 |
| *Missing* | *6* | *2* | *4* |  |
| 6 months |  |  |  |  |
| Average | 123.67 (117.67-130.67) | 122.33 (116.00-127.00) | 127.33 (121.00-131.67) | 0.38 |
| Change from baseline | -0.67 (-4.67-9.67) | 9.00 (-4.67-14.67) | -2.67 (-4.67--1.67) | 0.057 |
| *Missing* | *7* | *2* | *5* |  |
| 9 months |  |  |  |  |
| Average | 123.00 (117.67-128.67) | 121.00 (117.67-131.33) | 126.00 (116.33-128.67) | 0.57 |
| Change from baseline | -0.67 (-5.33-4.00) | 3.00 (-4.67-12.00) | -1.83 (-9.67-1.33) | 0.14 |
| *Missing* | *6* | *2* | *4* |  |
| 12 months |  |  |  |  |
| Average | 123.83 (118.50-130.00) | 119.67 (114.67-127.00) | 128.67 (120.00-134.00) | 0.081 |
| Change from baseline | 0.83 (-7.33-8.17) | 1.00 (-6.67-8.00) | 0.67 (-10.00-8.33) | 0.71 |
| *Missing* | *11* | *6* | *5* |  |
| Diastolic Blood Pressure across 3 sittings (mmHg) | |  |  |  |
| Baseline | 80.67 (73.67-86.00) | 73.67 (71.67-85.00) | 82.17 (79.00-86.00) | 0.099 |
| 3 months |  |  |  |  |
| Average | 79.33 (72.67-86.67) | 74.00 (68.33-88.00) | 80.17 (76.67-86.67) | 0.53 |
| Change from baseline | 0.33 (-3.67-2.33) | 0.33 (-0.67-8.33) | -0.83 (-6.67-2.33) | 0.42 |
| *Missing* | *6* | *2* | *4* |  |
| 6 months |  |  |  |  |
| Average | 81.83 (76.50-86.17) | 79.67 (76.33-86.33) | 83.00 (78.67-86.00) | 0.79 |
| Change from baseline | 0.17 (-4.67-6.00) | 3.33 (-4.67-11.67) | -2.33 (-4.67-0.00) | 0.13 |
| *Missing* | *7* | *2* | *5* |  |
| 9 months |  |  |  |  |
| Average | 79.00 (74.33-82.67) | 75.00 (66.00-84.33) | 79.00 (77.33-82.67) | 0.57 |
| Change from baseline | -0.67 (-5.00-2.00) | -0.33 (-3.67-8.33) | -2.67 (-5.67-1.67) | 0.26 |
| *Missing* | *6* | *2* | *4* |  |
| 12 months |  |  |  |  |
| Average | 79.50 (72.83-84.67) | 80.00 (69.00-87.33) | 79.33 (75.67-84.33) | 0.87 |
| Change from baseline | -2.00 (-5.00-3.33) | -1.00 (-2.00-11.33) | -5.00 (-6.00--2.00) | 0.044 |
| *Missing* | *11* | *6* | *5* |  |
| Total cholesterol (mmol/L) |  |  |  |  |
| Baseline | 4.40 (3.80-5.20) | 3.90 (3.60-4.40) | 4.90 (4.20-5.30) | 0.058 |
| 3 months |  |  |  |  |
| Average | 4.30 (3.80-5.00) | 3.90 (3.10-4.20) | 5.00 (4.40-5.40) | 0.006 |
| Change from baseline | 0.10 (-0.40-0.30) | 0.15 (-0.60-0.30) | 0.10 (0.10-0.20) | 0.62 |
| *Missing* | *8* | *3* | *5* |  |
| 6 months |  |  |  |  |
| Average | 4.60 (4.00-5.60) | 4.50 (3.60-4.70) | 4.85 (4.20-5.60) | 0.24 |
| Change from baseline | 0.20 (-0.30-0.60) | 0.40 (-0.30-0.70) | 0.15 (-0.45-0.50) | 0.60 |
| *Missing* | *4* | *2* | *2* |  |
| 9 months |  |  |  |  |
| Average | 4.60 (4.20-5.20) | 4.50 (4.20-4.90) | 4.85 (3.90-5.70) | 0.72 |
| Change from baseline | 0.50 (0.00-0.90) | 0.70 (0.30-1.20) | 0.30 (-0.20-0.90) | 0.29 |
| *Missing* | *6* | *2* | *4* |  |
| 12 months |  |  |  |  |
| Average | 4.90 (4.20-6.50) | 4.70 (3.60-6.70) | 5.25 (4.45-6.35) | 0.69 |
| Change from baseline | 0.40 (0.00-1.00) | 0.40 (0.10-1.00) | 0.55 (-0.55-1.15) | 0.73 |
| *Missing* | *12* | *6* | *6* |  |
| HDL-cholesterol (mmol/L) |  |  |  |  |
| Baseline | 1.20 (1.10-1.40) | 1.20 (1.15-1.40) | 1.26 (1.10-1.42) | 0.73 |
| 3 months |  |  |  |  |
| Average | 1.29 (1.03-1.48) | 1.07 (0.93-1.34) | 1.41 (1.20-1.48) | 0.041 |
| Change from baseline | 0.00 (-0.06-0.15) | -0.05 (-0.20-0.10) | 0.06 (0.00-0.16) | 0.12 |
| *Missing* | *8* | *3* | *5* |  |
| 6 months |  |  |  |  |
| Average | 1.37 (1.12-1.56) | 1.30 (1.12-1.56) | 1.40 (1.17-1.81) | 0.52 |
| Change from baseline | 0.10 (-0.10-0.29) | 0.10 (-0.10-0.29) | 0.14 (-0.04-0.29) | 0.67 |
| *Missing* | *4* | *2* | *2* |  |
| 9 months |  |  |  |  |
| Average | 1.40 (1.11-1.61) | 1.53 (1.18-1.70) | 1.39 (1.00-1.53) | 0.53 |
| Change from baseline | 0.20 (0.07-0.33) | 0.20 (0.10-0.39) | 0.14 (0.06-0.24) | 0.29 |
| *Missing* | *6* | *2* | *4* |  |
| 12 months |  |  |  |  |
| Average | 1.52 (1.30-1.77) | 1.32 (1.15-1.77) | 1.56 (1.35-1.85) | 0.52 |
| Change from baseline | 0.30 (0.10-0.49) | 0.30 (-0.10-0.34) | 0.33 (0.22-0.56) | 0.38 |
| *Missing* | *12* | *6* | *6* |  |
| LDL-cholesterol (mmol/L) |  |  |  |  |
| Baseline | 2.36 (1.60-2.95) | 1.90 (1.50-2.42) | 2.73 (1.60-3.03) | 0.14 |
| 3 months |  |  |  |  |
| Average | 1.90 (1.40-3.05) | 1.58 (1.20-2.20) | 2.95 (1.90-3.12) | 0.016 |
| Change from baseline | 0.03 (-0.50-0.24) | -0.10 (-0.50-0.24) | 0.10 (-0.08-0.21) | 0.41 |
| *Missing* | *8* | *3* | *5* |  |
| 6 months |  |  |  |  |
| Average | 2.40 (1.52-3.26) | 2.10 (1.40-2.84) | 2.68 (1.65-3.34) | 0.24 |
| Change from baseline | -0.03 (-0.29-0.39) | 0.20 (-0.29-0.44) | -0.06 (-0.20-0.38) | 0.95 |
| *Missing* | *4* | *2* | *2* |  |
| 9 months |  |  |  |  |
| Average | 2.10 (1.80-3.25) | 2.10 (1.80-2.57) | 2.43 (1.80-3.75) | 0.94 |
| Change from baseline | 0.20 (-0.01-0.61) | 0.22 (0.17-0.70) | 0.17 (-0.06-0.39) | 0.34 |
| *Missing* | *6* | *2* | *4* |  |
| 12 months |  |  |  |  |
| Average | 2.43 (1.96-4.07) | 2.10 (1.90-4.07) | 2.87 (2.28-4.08) | 0.35 |
| Change from baseline | 0.27 (-0.30-0.60) | 0.58 (-0.01-0.60) | 0.27 (-0.50-0.68) | 0.49 |
| *Missing* | *12* | *6* | *6* |  |
| Triglycerides (mmol/L) |  |  |  |  |
| Baseline | 1.61 (1.28-2.20) | 1.44 (1.21-1.99) | 1.70 (1.50-2.89) | 0.33 |
| 3 months |  |  |  |  |
| Average | 1.70 (1.40-2.40) | 1.60 (1.20-2.40) | 1.79 (1.44-2.20) | 0.51 |
| Change from baseline | 0.20 (-0.30-0.30) | 0.28 (-0.41-0.30) | -0.10 (-0.17-0.22) | 0.46 |
| *Missing* | *8* | *3* | *5* |  |
| 6 months |  |  |  |  |
| Average | 1.56 (1.10-2.30) | 1.67 (1.10-2.30) | 1.52 (1.11-2.20) | 0.76 |
| Change from baseline | -0.04 (-0.33-0.30) | 0.10 (-0.20-0.50) | -0.19 (-0.50-0.03) | 0.049 |
| *Missing* | *4* | *2* | *2* |  |
| 9 months |  |  |  |  |
| Average | 1.27 (1.19-2.33) | 1.70 (1.19-2.33) | 1.26 (1.20-2.50) | 0.81 |
| Change from baseline | -0.20 (-0.50-0.00) | -0.10 (-0.25-0.38) | -0.41 (-0.63--0.06) | 0.057 |
| *Missing* | *6* | *2* | *4* |  |
| 12 months |  |  |  |  |
| Average | 1.53 (1.00-1.97) | 1.90 (1.27-2.55) | 1.23 (0.94-1.75) | 0.13 |
| Change from baseline | -0.21 (-0.67--0.01) | -0.01 (-0.30-0.20) | -0.55 (-0.69--0.15) | 0.037 |
| *Missing* | *12* | *6* | *6* |  |

Missing data is indicated. Data for all timepoints is reported as Median (Interquartile Range) due to a number of non-normally distributed variables. Differences between treatment groups were examined using Wilcoxon rank-sum test.

**Supplementary Table 13: Impact of Weight on Quality of Life (IWQoL) measures across follow-up**

|  | Total (n=27) | Liraglutide (n=13) | Placebo (n=14) | p-value |
| --- | --- | --- | --- | --- |
| IWQoL lite - Physical function | |  |  |  |
| Baseline | 30.0 (23.0-33.0) | 32.0 (25.0-37.0) | 28.5 (23.0-32.0) | 0.19 |
| 3 months |  |  |  |  |
| Average | 24.0 (18.0-28.0) | 25.00 (18.0-35.0) | 22.00 (19.0-27.5) | 0.56 |
| Change from baseline | -6.0 (-10.0--2.0) | -8.0 (-11.0--5.0) | -4.5 (-7.0--0.5) | 0.079 |
| *Missing* | *4* | *2* | *2* |  |
| 6 months |  |  |  |  |
| Average | 21.0 (16.0-30.0) | 24.00 (18.5-36.5) | 16.0 (13.0-23.0) | 0.036 |
| Change from baseline | -7.0 (-11.0--1.0) | -6.5 (-11.0-2.0) | -7.0 (-12.0--4.0) | 0.46 |
| *Missing* | *4* | *1* | *3* |  |
| 12 months |  |  |  |  |
| Average | 20.0 (16.0-27.0) | 22.00 (18.0-35.0) | 18.50 (14.0-26.0) | 0.25 |
| Change from baseline | -6.0 (-12.0--3.0) | -6.0 (-9.0--2.0) | -5.5 (-14.0--4.0) | 0.46 |
| *Missing* | *8* | *4* | *4* |  |
| IWQoL lite - Self esteem |  |  |  |  |
| Baseline | 22.0 (15.0-29.0) | 27.0 (18.0-29.0) | 21.0 (14.0-28.0) | 0.71 |
| 3 months |  |  |  |  |
| Average | 19.0 (13.0-22.0) | 19.0 (14.0-27.0) | 19.00 (13.0-20.5) | 0.32 |
| Change from baseline | -3.0 (-7.0-0.0) | -4.0 (-6.0--3.0) | 0.00 (-9.0-1.5) | 0.31 |
| *Missing* | *4* | *2* | *2* |  |
| 6 months |  |  |  |  |
| Average | 17.0 (9.0-25.0) | 20.5 (12.0-27.0) | 12.0 (8.0-19.0) | 0.12 |
| Change from baseline | -4.0 (-8.0-0.0) | -4.0 (-7.0--0.5) | -5.0 (-14.0-0.0) | 0.54 |
| *Missing* | *4* | *1* | *3* |  |
| 12 months |  |  |  |  |
| Average | 16.5 (10.5-21.0) | 19.0 (16.0-27.0) | 13.0 (10.0-17.0) | 0.17 |
| Change from baseline | -3.5 (-6.5--1.5) | -3.0 (-5.0-0.0) | -5.0 (-14.0--3.0) | 0.16 |
| *Missing* | *7* | *4* | *3* |  |
| IWQoL lite - Sexual life |  |  |  |  |
| Baseline | 12.0 (8.0-18.0) | 13.0 (12.0-20.0) | 10.5 (8.0-15.0) | 0.29 |
| 3 months |  |  |  |  |
| Average | 9.0 (4.5-15.5) | 11.5 (8.0-16.0) | 5.5 (4.0-9.0) | 0.067 |
| Change from baseline | -1.0 (-4.0-0.0) | -1.0 (-3.0-0.0) | -1.0 (-5.0-0.0) | 0.42 |
| *Missing* | *7* | *3* | *4* |  |
| 6 months |  |  |  |  |
| Average | 9.0 (4.0-15.0) | 13.0 (4.0-16.0) | 6.5 (4.0-11.0) | 0.19 |
| Change from baseline | -2.0 (-4.0-0.0) | 0.0 (-4.0-0.0) | -2.0 (-4.0-0.0) | 0.56 |
| *Missing* | *8* | *4* | *4* |  |
| 12 months |  |  |  |  |
| Average | 8.0 (4.0-13.0) | 12.5 (4.0-14.0) | 8.0 (5.0-10.0) | 0.44 |
| Change from baseline | 0.0 (-4.0-0.0) | 0.0 (-4.0-0.0) | -1.0 (-4.0-0.0) | 0.50 |
| *Missing* | *12* | *7* | *5* |  |
| IWQoL lite - Public distress | |  |  |  |
| Baseline | 13.0 (9.0-15.0) | 13.0 (11.0-15.0) | 13.5 (9.0-15.0) | 0.88 |
| 3 months |  |  |  |  |
| Average | 9.5 (5.5-13.0) | 9.0 (7.5-13.0) | 9.50 (5.0-12.5) | 0.64 |
| Change from baseline | -2.0 (-5.0-0.0) | -1.5 (-5.0-0.0) | -2.00 (-3.5-0.0) | 0.77 |
| *Missing* | *3* | *1* | *2* |  |
| 6 months |  |  |  |  |
| Average | 7.0 (5.0-12.0) | 7.0 (5.0-14.0) | 6.00 (5.0-11.0) | 0.28 |
| Change from baseline | -3.0 (-5.0-0.0) | -3.0 (-5.0-0.0) | -4.00 (-6.0-0.0) | 0.82 |
| *Missing* | *5* | *2* | *3* |  |
| 12 months |  |  |  |  |
| Average | 5.0 (5.0-11.0) | 6.5 (5.0-11.5) | 5.0 (5.0-11.0) | 0.40 |
| Change from baseline | -5.0 (-7.0-0.0) | -3.5 (-7.5-0.0) | -5.0 (-7.0--2.0) | 0.62 |
| *Missing* | *8* | *5* | *3* |  |
| IWQoL lite - Work |  |  |  |  |
| Baseline | 9.00 (5.00-11.00) | 9.00 (7.00-11.00) | 8.50 (5.00-11.0) | 0.94 |
| 3 months |  |  |  |  |
| Average | 7.5 (4.0-9.0) | 7.50 (5.0-9.0) | 7.0 (4.0-9.0) | 0.84 |
| Change from baseline | -2.5 (-3.5-1.0) | -1.50 (-3.5-1.0) | -2.5 (-3.5-0.00 | 0.75 |
| *Missing* | *3* | *1* | *2* |  |
| 6 months |  |  |  |  |
| Average | 6.0 (4.0-10.0) | 7.0 (4.0-10.0) | 5.0 (4.0-10.0) | 0.53 |
| Change from baseline | -1.0 (-4.0-0.0) | -1.0 (-2.0-0.0) | -2.0 (-6.0-1.0) | 0.62 |
| *Missing* | *6* | *3* | *3* |  |
| 12 months |  |  |  |  |
| Average | 5.0 (4.0-8.0) | 5.5 (4.0-9.5) | 5.00 (4.0-8.0) | 0.57 |
| Change from baseline | -1.0 (-5.0-0.0) | 0.0 (-2.0-0.5) | -2.00 (-6.0-0.0) | 0.19 |
| *Missing* | *8* | *5* | *3* |  |
| IWQoL - Total Score |  |  |  |  |
| Baseline | 92.0 (74.0-103.0) | 96.0 (90.0-99.0) | 81.5 (57.0-103.0) | 0.48 |
| 3 months |  |  |  |  |
| Average | 68.0 (51.0-86.0) | 75.00 (62.0-88.0) | 54.50 (49.0-78.0) | 0.22 |
| Change from baseline | -8.0 (-25.0--3.0) | -15.0 (-23.0--8.0) | -5.5 (-37.0-2.0) | 0.25 |
| *Missing* | *8* | *4* | *4* |  |
| 6 months |  |  |  |  |
| Average | 61.5 (41.0-81.0) | 77.5 (61.5-88.5) | 45.5 (36.0-69.0) | 0.075 |
| Change from baseline | -14.0 (-24.0--10.0) | -16.0 (-20.0--9.5) | -12.0 (-42.0--10.0) | 0.76 |
| *Missing* | *9* | *5* | *4* |  |
| 12 months |  |  |  |  |
| Average | 58.0 (40.0-73.0) | 71.5 (55.0-83.0) | 47.5 (39.5-67.0) | 0.25 |
| Change from baseline | -18.0 (-32.0--13.0) | -16.0 (-19.0--9.0) | -24.5 (-50.5--16.5) | 0.19 |
| *Missing* | *13* | *7* | *6* |  |

Missing data is indicated. Data for all timepoints is reported as Median (Interquartile Range) due to a number of non-normally distributed variables. Differences between treatment groups were examined using Wilcoxon rank-sum test. IWQoL = Impact of Weight on Quality of Life.

**Supplementary Table 14: EQ-5D-5L measures across follow-up**

|  | Total (n=27) | Liraglutide (n=13) | Placebo (n=14) | p-value |
| --- | --- | --- | --- | --- |
| EQ-5D-5L Utility Index |  |  |  |  |
| Baseline | 0.81 (0.74-1.00) | 0.81 (0.74-1.00) | 0.84 (0.74-1.00) | 0.73 |
| 3 months |  |  |  |  |
| Average | 0.87 (0.81-1.00) | 0.82 (0.74-1.00) | 0.93 (0.85-0.94) | 0.25 |
| Change from baseline | 0.00 (-0.08-0.11) | 0.00 (-0.08-0.07) | -0.00 (-0.07-0.19) | 0.58 |
| 6 months |  |  |  |  |
| Average | 0.94 (0.87-1.00) | 0.87 (0.81-1.00) | 1.00 (0.93-1.00) | 0.023 |
| Change from baseline | 0.00 (0.00-0.13) | 0.00 (-0.06-0.02) | 0.05 (0.00-0.19) | 0.065 |
| 12 months |  |  |  |  |
| Average | 0.90 (0.81-1.00) | 0.90 (0.57-1.00) | 0.91 (0.84-1.00) | 0.49 |
| Change from baseline | 0.00 (-0.11-0.12) | -0.07 (-0.11-0.00) | 0.03 (-0.06-0.12) | 0.21 |
| EQ Visual Analogue Scale | |  |  |  |
| Baseline | 80.0 (65.0-85.0) | 77.5 (65.0-90.0) | 80.0 (67.5-85.0) | 0.95 |
| *Missing* | *3* | *1* | *2* |  |
| 3 months |  |  |  |  |
| Average | 75.0 (60.0-90.0) | 70.0 (55.0-87.5) | 79.0 (70.0-95.0) | 0.25 |
| Change from baseline | 0.0 (-12.5-7.5) | 0.0 (-15.0-5.0) | -2.0 (-10.0-10.0) | 0.91 |
| *Missing* | *4* | *1* | *3* |  |
| 6 months |  |  |  |  |
| Average | 80.0 (70.0-90.0) | 70.0 (65.0-95.0) | 85.0 (75.0-90.0) | 0.44 |
| Change from baseline | 0.0 (-5.0-8.0) | 0.0 (-5.0-5.0) | 0.0 (-5.0-10.0) | 0.88 |
| *Missing* | *6* | *2* | *4* |  |
| 12 months |  |  |  |  |
| Average | 80.0 (70.0-90.0) | 76.0 (55.0-90.0) | 82.0 (72.5-92.5) | 0.13 |
| Change from baseline | 0.0 (-10.0-5.0) | -10.0 (-10.0-5.0) | 0.0 (-10.0-5.0) | 0.41 |
| *Missing* | *8* | *4* | *4* |  |

Missing data is indicated. Data for all timepoints is reported as Median (Interquartile Range) due to a number of non-normally distributed variables. Differences between treatment groups were examined using Wilcoxon rank-sum test. EQ-5D-5L = self-assessed health related, quality of life questionnaire

**Supplementary Table 15: Hospital Anxiety and Depression Scale (HADS) across follow-up**

|  | Total (n=27) | Liraglutide (n=13) | Placebo (n=14) | p-value |
| --- | --- | --- | --- | --- |
| HADS Anxiety Score |  |  |  |  |
| Baseline | 9.0 (5.0-11.0) | 8.0 (5.0-10.0) | 9.0 (4.0-12.0) | 0.72 |
| *Missing* | *2* | *2* | *0* |  |
| 6 months |  |  |  |  |
| Average | 6.0 (5.0-9.0) | 7.5 (5.0-12.0) | 5.00(4.0-8.0) | 0.099 |
| Change from baseline | -1.5 (-3.0-3.0) | -1.0 (-3.0-4.0) | -2.0 (-3.0-0.0) | 0.20 |
| *Missing* | *4* | *1* | *3* |  |
| 12 months |  |  |  |  |
| Average | 8.0 (3.0-10.0) | 8.50 (6.0-11.0) | 5.0 (2.0-9.0) | 0.31 |
| Change from baseline | -2.5 (-4.0-0.0) |  | -3.0 (-7.0-0.0) | 0.59 |
| *Missing* | *6* | *3* | *3* |  |
| HADS Depression Score |  |  |  |  |
| Baseline | 7.0 (3.0-9.0) | 7.5 (3.0-9.5) | 6.5 (2.0-9.0) | 0.60 |
| *Missing* | *1* | *1* | *0* |  |
| 6 months |  |  |  |  |
| Average | 5.0 (2.0-8.0) | 7.5 (3.5-9.0) | 2.0 (0.0-6.0) | 0.067 |
| Change from baseline | -2.0 (-3.0-0.0) | -1.0 (-3.0-0.0) | -2.0 (-4.0--2.0) | 0.14 |
| *Missing* | *4* | *1* | *3* |  |
| 12 months |  |  |  |  |
| Average | 6.0 (2.0-8.0) | 7.0 (1.5-9.0) | 4.5 (2.0-6.0) | 0.44 |
| Change from baseline | -1.0 (-4.0-2.0) | -1.0 (-2.0-2.0) | -1.5 (-5.0-1.0) | 0.49 |
| *Missing* | *9* | *5* | *4* |  |

Missing data is indicated. Data for all timepoints is reported as Median (Interquartile Range) due to a number of non-normally distributed variables. Differences between treatment groups were examined using Wilcoxon rank-sum test. N = number, HADS = Hospital Anxiety and Depression Scale

**Supplementary Table 16: Gastric Band Adjustments**

|  | Total (n=27) | Liraglutide (n=13) | Placebo (n=14) | p-value |
| --- | --- | --- | --- | --- |
| Total number of band adjustments | 3.0 (2.0-4.0) | 3.0 (2.0-5.0) | 3.0 (2.0-3.0) | 0.39 |
| Time-specific adjustments |  |  |  |  |
| Randomisation to 6 months | 2.0 (2.0-3.0) | 2.5 (1.0-3.0) | 2.0 (2.0-2.5) | 0.75 |
| 6 to 12 months | 1.0 (1.0-2.0) | 1.0 (1.0-2.5) | 1.0 (0.0-1.0) | 0.16 |

Number of band adjustments is reported as Median (Interquartile Range) due to a non-normal distribution. Differences between treatment groups were examined using Wilcoxon rank-sum test.

## Supplementary Table 17: Site Specific Analysis for HbA1c and Body Weight

## Supplementary Table 17.1: Site specific analysis of HbA1c

|  | Total (n=27) | GSTT (n=16) | BHH (n=4) | NB (n=7) | p-value |
| --- | --- | --- | --- | --- | --- |
| Screening | 57.0 (48.0-65.0) | 54.0 (48.5-71.5) | 62.0 (60.0-68.0) | 54.0 (44.0-61.0) | 0.26 |
| Baseline | 51.0 (43.0-63.0) | 48.5 (41.5-64.5) | 58.0 (56.5-68.5) | 51.0 (43.0-54.0) | 0.29 |
| 3 months |  |  |  |  |  |
| Average HbA1c (mmol/mol) | 52.5 (43.0-80.5) | 46.5 (41.0-75.0) | 65.0 (55.0-94.0) | 50.0 (45.0-86.0) | 0.39 |
| Change from baseline | -0.5 (-4.0-7.5) | -3.5 (-9.0-1.0) | 8.0 (-1.0-35.0) | 3.0 (-4.0-31.0) | 0.10 |
| *Missing* | *7* | *6* | *1* | *0* |  |
| 6 months |  |  |  |  |  |
| Average HbA1c | 49.0 (42.0-63.0) | 43.5 (39.0-52.0) | 61.0 (55.0-66.5) | 61.0 (43.0-78.0) | 0.11 |
| Change from baseline | -3.0 (-11.0-3.0) | -6.0 (-15.0-2.0) | 0.5 (-12.0-8.5) | 8.0 (-1.0-18.0) | 0.021 |
| *Missing* | *2* | *2* | *0* | *0* |  |
| 9 months |  |  |  |  |  |
| Average HbA1c (mmol/mol) | 51.0 (44.0-67.0) | 49.0 (43.0-53.0) | 77.0 (70.0-84.0) | 50.0 (46.0-67.0) | 0.16 |
| Change from baseline | 1.0 (-5.0-6.0) | 0.0 (-10.0-1.0) | 19.0 (13.0-25.0) | 3.0 (1.0-16.0) | 0.023 |
| *Missing* | *6* | *3* | *2* | *1* |  |
| 12 months |  |  |  |  |  |
| Average HbA1c (mmol/mol) | 54.5 (45.0-63.0) | 50.5 (43.0-56.0) | 67.0 (65.0-70.0) | 57.0 (51.0-59.0) | 0.053 |
| Change from baseline | 5.5 (-7.0-8.0) | 2.5 (-11.0-8.0) | 8.0 (-13.0-13.0) | 6.0 (4.0-8.0) | 0.72 |
| *Missing* | *9* | *6* | *1* | *2* |  |

Variables with missing data are indicated. All variables are reported as Median (Interquartile Range) due to a number of non-normally distributed variables. Differences between treatment groups were examined using Kruskal-Wallis test.

## Supplementary Table 17.2: Site specific analysis of Body Weight

|  | Total (n=27) | GSTT (n=16) | BHH (n=4) | NB (n=7) | p-value |
| --- | --- | --- | --- | --- | --- |
| Screening | 107.4 (97.8-127.5) | 105.7 (99.4-110.9) | 84.1 (81.8-104.8) | 145.8 (125.0-152.4) | 0.001 |
| Baseline | 102.0 (91.7-123.3) | 98.9 (92.8-105.1) | 80.1 (76.5-98.6) | 140.0 (119.2-143.5) | 0.001 |
| 3 months |  |  |  |  |  |
| Average Weight (kg) | 101.7 (87.3-122.2) | 90.5 (86.7-103.0) | 79.4 (79.1-116.2) | 136.5 (119.9-146.5) | 0.003 |
| Change from baseline | -1.8 (-4.1-0.3) | -3.8 (-6.4--2.0) | 0.4 (0.2-5.1) | 0.7 (-1.5-3.0) | 0.002 |
| *Missing* | *3* | *2* | *1* | *0* |  |
| 6 months |  |  |  |  |  |
| Average Weight (kg) | 92.7 (86.6-121.2) | 87.3 (86.6-93.4) | 78.3 (78.2-78.3) | 140.3 (121.2-154.3) | 0.47 |
| Change from baseline | -2.8 (-7.7-2.0) | -7.6 (-10.0--2.9) | 1.8 (-0.7-4.2) | 2.0 (0.3-5.6) | 0.92 |
| *Missing* | *5* | *3* | *2* | *0* |  |
| 9 months |  |  |  |  |  |
| Average Weight (kg) | 95.7 (82.4-118.5) | 90.0 (86.5-113.0) | 78.3 (73.0-79.4) | 135.9 (119.0-142.2) | 0.49 |
| Change from baseline | -1.5 (-7.3-0.4) | -4.1 (-12.6-0.3) | -0.7 (-8.2-5.4) | -0.8 (-2.9-6.7) | 0.45 |
| *Missing* | *5* | *3* | *1* | *1* |  |
| 12 months |  |  |  |  |  |
| Average Weight (kg) | 88.9 (80.4-121.0) | 87.5 (80.9-106.7) | 78.3 (75.6-96.9) | 135.6 (134.1-142.0) | 0.16 |
| Change from baseline | -4.4 (-9.4-1.7) | -8.5 (-13.0-1.3) | -0.6 (-4.5-1.8) | -3.3 (-4.4-5.0) | 0.067 |
| *Missing* | *6* | *4* | *0* | *2* |  |

Variables with missing data are indicated. All variables are reported as Median (Interquartile Range) due to a number of non-normally distributed variables. Differences between treatment groups were examined using Kruskal-Wallis test.

**Supplementary Clinical Data 1: Missing HbA1c data**

**Missing HbA1c Data:** Overall, 8 patients (4 in each treatment group) exhibited monotonic missingness – where data is missing and one timepoint and all subsequent timepoints. This includes the 2 patients who only have baseline HbA1c measures. An additional 5 patients (2 on Liraglutide, 3 on Placebo) had missing data for random timepoints but have complete data for later timepoints. All patients except the two who with no follow-up, have HbA1c measured at 6 months.

**Missing Weight Data:** The 2 patients with only baseline weight recorded are the same patients with only baseline HbA1c recorded. Other patients with missing data sometimes differed – either missing difference timepoints or didn’t/did have missing data. Overall, 6 patients (3 in each treatment group) had monotonic missingness - including the 2 with only baseline data. 4 patients (1 on Liraglutide, 3 on Placebo) had missing data for missing data for random timepoints.

**Supplementary Clinical Sequelae 1: Hypoglycaemic events**

Only one recorded hypoglycaemic event occurred post-randomisation. A patient in the placebo arm experienced a hypoglycaemic event 2 days after randomisation that was classed as not serious, was self-treated by the patient. Blood glucose during the event is recorded at 4.7mmol/L.

**Supplementary Results Description 1**

**Anthropometric parameters**

Anthropometric measurements at 6 and 12 months showed no significant change from randomisation for BMI (6 months: L -1.0, P-1.0, p = 0.92; 12 months: L 0.4, P -2.9, p = 0.14), neck circumference (6m: L -1.0cm, P -1.0, p = 0.8; 12m: L 0.0cm, P -2.0, p = 0.36), waist circumference (6m: L -4.0cm, P -6.0, p = 0.68, 12m: L 3.0cm, P: -10.0, p = 0.16), fat-free mass (6m: L -1.3kg, P -0.7, p = 0.77; 12m: L 0.3kg, P -3.3kg, p = 0.14) and body fat percentage (6m: L 0.2%, P -2.2, p = 0.54, 12m: L -0.6%, P -1.7, p = 0.14). There was a significant reduction in fat mass (6m: L -1.3kg, P -4.9, p = 0.5; 12m: L 1.3kg, P -7.2, p = 0.035) at 12 months in the placebo group, but this was not shown at 6 months (**Supplementary Table 11**).

**Cardiovascular disease risk factors**

There was no significant difference between groups at 6 and 12 months from randomisation in total cholesterol (6m: L 0.4mmol/l, P 0.2, p = 0.60, 12m L 0.4, P 0.6, p = 0.73), HDL-cholesterol (6m: L 0.1mmol/l, P 0.1, p = 0.67; 12m: L 0.3, P 0.3, p = 0.38) or LDL-cholesterol (6m: L 0.2mmol/l, P -0.1, p = 0.95; 12m L 0.6, P 0.3, p = 0.49). Systolic blood pressure trended towards being lower in the placebo group at 6 months (L 9.0mmHg, P -2.7, p = 0.057), but this trend did not continue to 12 months (L 1.0mmHg, P 0.7, p = 0.71). Diastolic blood pressure was significantly lower at 12 months (L – 1.0mmHg, P -5.0, p = 0.044) but not at 6 months (L 3.3mmHg, P -2.3, p = 0.13). Triglyceride levels reduced significantly more in placebo at 6 and 12 months (6m: L 0.1mmol/l, P -0.2, p = 0.049, 12m: L -0.0, P -0.6, p = 0.037) (**Supplementary Table 12**).

**Quality of Life**

There was no significant change in quality-of-life (IWQoL,, EQ-5D-5L, HADS) measures throughout the study (**Supplementary Table 13-15GLID**).
